# Altered phase and nonphase EEG activity expose impaired maintenance of a spatial-object attentional focus in multiple sclerosis patients

**DOI:** 10.1038/s41598-020-77690-y

**Published:** 2020-11-26

**Authors:** M. Vazquez-Marrufo, E. Sarrias-Arrabal, R. Martin-Clemente, A. Galvao-Carmona, G. Navarro, G. Izquierdo

**Affiliations:** 1 Lab B508 (Psychophysiology Unit), Experimental Psychology Department, Faculty of Psychology, University of Seville, Seville, Spain; 2 Signal Processing and Communications Department, Higher Technical School of Engineering, University of Seville, Seville, Spain; 3 Department of Psychology, Universidad Loyola Andalucía, Seville, Spain; 4 Multiple Sclerosis Unit, Hospital Universitario Virgen Macarena, Seville, Spain; 5 Multiple Sclerosis Unit, Hospital Vithas, Seville, Spain

**Keywords:** Neuroscience, Psychology

## Abstract

Some of the anatomical and functional basis of cognitive impairment in multiple sclerosis (MS) currently remains unknown. In particular, there is scarce knowledge about modulations in induced EEG (nonphase activity) for diverse frequency bands related to attentional deficits in this pathology. The present study analyzes phase and nonphase alpha and gamma modulations in 26 remitting-relapsing multiple sclerosis patients during their participation in the attention network test compared with twenty-six healthy controls (HCs) matched in sociodemographic variables. Behavioral results showed that the MS group exhibited general slowing, suggesting impairment in alerting and orienting networks, as has been previously described in other studies. Time–frequency analysis of EEG revealed that the gamma band was related to the spatial translation of the attentional focus, and the alpha band seemed to be related to the expectancy mechanisms and cognitive processing of the target. Moreover, phase and nonphase modulations differed in their psychophysiological roles and were affected differently in the MS and HC groups. In summary, nonphase modulations can unveil hidden cognitive mechanisms for phase analysis and complete our knowledge of the neural basis of cognitive impairment in multiple sclerosis pathology.

## Introduction

Multiple sclerosis (MS) is a chronic degenerative neurological disease of unknown etiology that causes mainly neurological damage including demyelination and inflammation of the central nervous system (CNS)^1^. The onset of MS occurs over a wide age range (mainly between 20 and 40 years), and MS predominantly affects women. Approximately 40–70% of patients with the disease show diverse cognitive deficits^1–3^. Therefore, different neuropsychological profiles can be found during clinical assessments. Attention, processing speed and memory are the cognitive domains most frequently affected^4–7^. To understand the neural basis of these cognitive alterations in MS, different cognitive paradigms and techniques have been used^8–10^.

One of the cognitive paradigms that has often been used to evaluate attention is the “Attention Network Test” (ANT). The ANT was developed to assess three attentional networks (alerting, orienting and executive systems) and has been performed with different versions and for diverse pathologies^11–14^.

Although the ANT was developed in 2001^15^, it did not begin to be applied in patients with multiple sclerosis until 2010^16^. These authors found an alteration in patients’ alerting network with respect to healthy controls (HCs). The authors suggested that the deficit could be due to lower response readiness or a reduced ability of patients to maintain alertness after presentation of the cue. Other authors have confirmed the alteration in the alerting network in patients with multiple sclerosis even in early stages of the disease^17^. Moreover, a study^18^ found deficits in the alerting and orienting networks in patients based not only on behavioral responses but also on specific psychophysiological variables (e.g., changes in the amplitude of the contingent negative variation).

One way to improve our knowledge of the neural basis of cognitive impairment in MS pathology is to analyze the frequency domain of the EEG signal. In its origins, the EEG signal was largely studied using fast Fourier transformation (FFT), but this approach has the disadvantage of renouncing the high EEG time resolution^19,20^. Time–frequency EEG methods such as temporal spectral evolution (TSE) can overcome this limit^21^. TSE shows EEG time–frequency information that includes both phase (evoked) and nonphase (induced) activity related to stimulus presentation (for a detailed description of the TSE method^22,23^).

Through TSE and other time–frequency techniques, numerous studies have related frequency bands of EEG to specific cognitive mechanisms. In the case of the alpha band, originally, it was identified as an indicator of a neural area at rest^24^. However, some authors have argued that it may not exclusively reflect an idling status but can also play an active role in inhibitory control and timing of sensory processing^25^. Along this line, some authors have suggested that an increase in alpha activity in regions irrelevant to the task may reflect inhibition processes^26^. Specifically, for attentional visual processing, some studies have observed higher alpha activity in those regions of the occipital cortex where unattended locations are retinotopically represented^27–29^.

For decades, it has been suggested that there are two types of alpha bands, namely, low alpha (8–10.5 Hz) and upper alpha (10.5–13 Hz) bands, with different psychophysiological meanings^30^. Low alpha bands may be related to a general attention, while upper alpha bands are proposed to be linked to more specific processes such as semantic processing^31,32^. In fact, the topographical distribution of low alpha bands is usually larger and less specific than that of upper alpha bands, which tends to be more localized in scalp regions^33,34^.

Another neurophysiological correlate that has been linked to cognitive functioning is the gamma band^35,36^. The functional roles assigned to this band are more than the visual grouping, as has been reviewed by some authors^37^. In particular, this neural activity has been related to arousal or attention in humans^38–40^. One of the reasons that this band is suitable for attentional mechanisms is its fine temporal tuning for neuronal firing (10–30 ms time precision)^41^. Some studies have described the specific involvement of the gamma band in visual attentional tasks^41,42^. In the study by Gruber et al.^41^, the authors displayed colored rectangles in the left or right half of the screen, and a cue indicated to the subjects in which visual hemifield they had to shift their attention to detect target stimuli. These authors found an increase in the gamma activity (35–51 Hz) when the attended stimuli were rotating. In the study by Fries et al.^42^, neurons from cortical area V4 in macaque monkeys exhibited an increase in the gamma band (35–90 Hz) when the experimental subjects were attending to relevant stimuli and ignoring distracters.

With regard to these spectral bands and their relation to cognitive impairment in MS, some studies have described a higher decrement of the alpha band in MS patients reflecting a recruitment of more cognitive resources to perform the task^43^. A more recent study^44^ showed that the alpha band is related to attentional mechanisms and processing speed; a smaller decrease in the resting-state alpha band in specific experimental conditions was associated with a poorer behavioral performance.

With regard to the gamma band, a few studies have described alterations in this band in MS patients related to diverse phenomena, such as alterations in cognitive processing in a visual attention task^45^, a neurophysiological correlate of compensatory mechanisms or cortical excitatory states representative of some phases during the MS course^45^, or gray matter dysfunction, possibly mediated by GABAergic changes^46^.

Finally, there is scarce knowledge about the meaning of the spectral modulations observed during the execution of the ANT^47–49^. Authors have described a decrease in the alpha band after the warning signal (alerting effect) and an increase in the gamma content at 200 ms after target onset related to the orienting network^47^. However, this study did not consider if the modulation was in phase or nonphase to the onset of the stimuli^47^. In the last years, neuroscientists have focused on this difference and found that both domains (evoked and induced) could represent diverse processes in cognition^50–52^.

Considering all these premises, the main goal of the present study was to analyze the modulations in the alpha and gamma bands (and in both evoked and induced modalities) related to attentional impairment in an MS group assessed by the ANT. Our hypothesis was that behavioral data would show a general slowing in the MS group compared to the healthy group that would be corroborated by the neuropsychological assessment. Alpha and gamma modulations related to attentional mechanisms would show alterations in the MS group during the execution of the ANT. An open question in the present study was which components of the alpha and gamma modulations (evoked, induced or both) would be related to attentional impairment.

## Results

### Behavioral data

The HCs obtained a significantly shorter reaction time than the MS group [F (1,50) = 26,55; *p* < 0.001; η^2^ = 0.438]. In addition, the cue factor revealed differences between cues, with the no cue (NC) condition showing larger reaction times followed by the central cue (CC) condition and finally the spatial cue (SC) condition, which was the fastest [F (2,100) = 287.04; *p* < 0.001; η^2^ = 0.930] (Table 1and supplementary Table 1 for statistical results). Post hoc Bonferroni comparisons confirmed the differences between all cue levels (*p* < 0.001).

**Table 1:** ANT behavioral results.

Reaction time (mean ± SD)							Accuracy (mean ± SD)	
Conditions	Correction not applied		*P* value*	Correction applied		*P*-value**	MS	HC
	MS	HC		MS	HC			
NC	638 ± 81	531 ± 71	< 0.001	1.05 ± 0.02	1.06 ± 0.01	0.03	92.90 ± 11.21	96.51 ± 2.49
CC	614 ± 85	505 ± 74	< 0.001	1.01 ± 0.02	1.00 ± 0.01	0.72	94.07 ± 9.73	96 ± 3.86
SC	568 ± 78	462 ± 63	< 0.001	0.93 ± 0.02	0.92 ± 0.02	0.12	95.35 ± 5.26	96.78 ± 2.43
Mean	607 ± 80	500 ± 69	< 0.001	–	–	–	94.11 ± 8.52	96.42 ± 2.39

*The *p* value refers to the ‘‘Group’’ factor before correction for general slowing had been applied (see text for details). **The *P* value refers to the “Group” factor after correction for general slowing had been applied. Abbreviations: MS: multiple sclerosis; HC: healthy controls. SD: standard deviation, RT: reaction time, NC: no cue; CC: central cue; SC: spatial cue.

After applying the correction of Fernández-Duque and Black^53^, differences in the NC condition were observed between groups [t = 2.186; *p* = 0.03], and patients were faster than HCs. This value does not indicate that MS patients were faster than HC subjects in this condition; it shows that MS patients were relatively faster for this condition than in all conditions in the MS group in the same comparison for HCs.

With regard to precision, there were no significant differences between groups or other factors because of a similar high percentage of accuracy for all conditions in both experimental groups (Table 1).

### Neuropsychological and depression assessments

In terms of the number of hits on the PASAT-3s and the overall hit rate on the symbol digit modality test (SDMT), the latter showed a value of 2 SD under the cutoff scores defined by Sepulcre^54^, indicating a moderate attentional impairment in our sample of MS patients (SDMT score = 44 ± 13.82). Last, Beck depression scores (BDI-II) indicated that there were not severe signs of depression in the MS group (BDI-II score = 7.44 ± 6.3).

### Gamma activity

#### Expectancy interval

**Figure 1:**
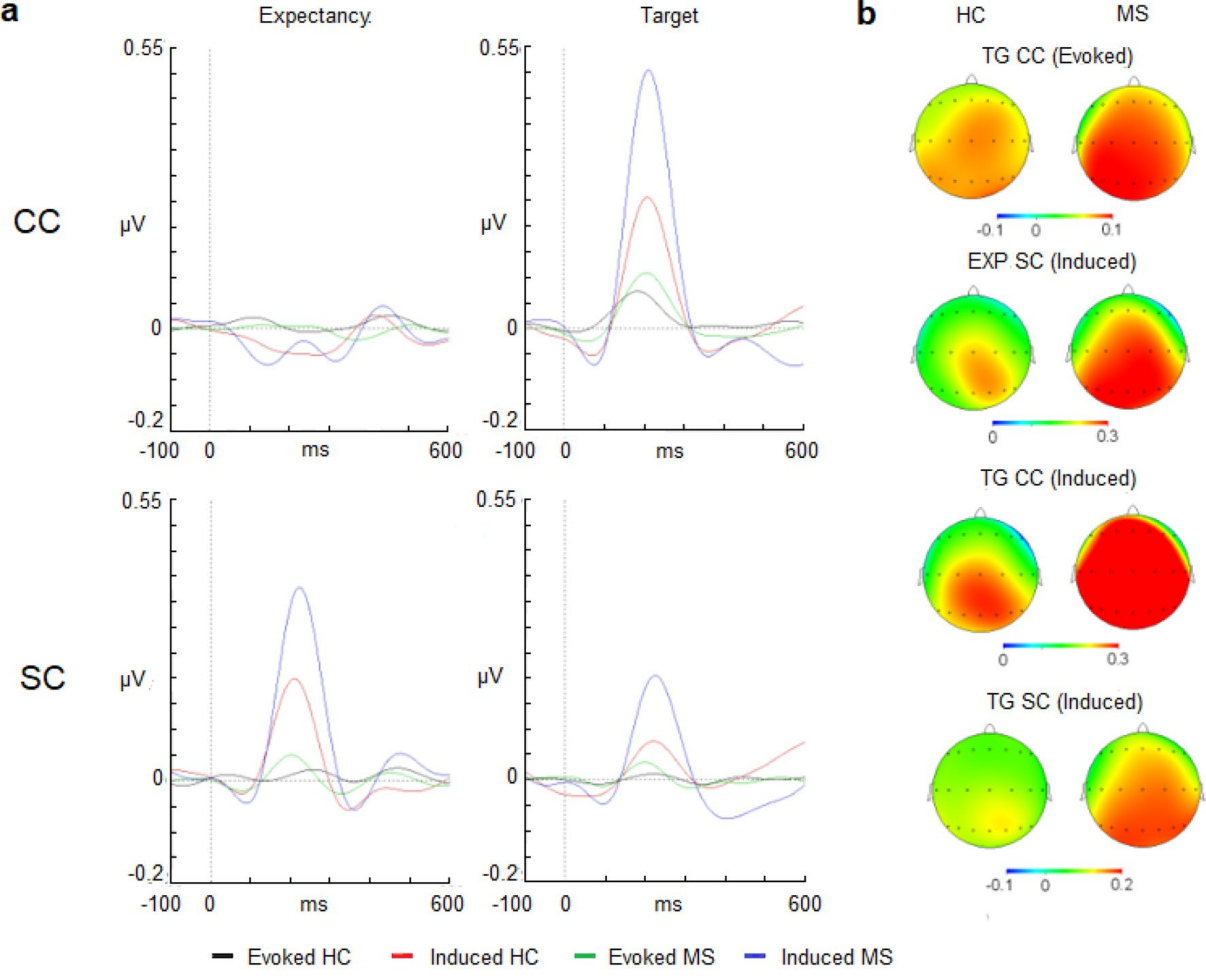
(**a**) Evoked and induced waves for the gamma band (30–45 Hz) in the expectancy and target response intervals. (**b**) 2-D head maps for the peak and valley latencies of evoked and induced activity. Abbreviations: MS: multiple sclerosis; HC: healthy controls; CC: central cue; SC: spatial cue; EXP: expectancy interval; ms: milliseconds.

The evoked gamma activity showed no differences in latency after presentation of an SC between both groups (Fig. 1).There were no appreciable modulations for the NC and CC conditions to calculate the latency. With regard to the amplitude, the evoked gamma activity showed no differences after presentation of any type of cue (NC, CC or SC) or between both groups (Fig. 1 and Table 2).

**Table 2:** Evoked and induced values for the gamma band (30–45 Hz).

Latency (mean ± SD)					
Expectancy interval			Target interval		
Conditions	MS	HC	Conditions	MS	HC
**Evoked activity**			**Evoked activity**		
**NC**	–	–	**NC**	199 ± 55	220 ± 53
**CC**	–	–	**CC**	209 ± 46	186 ± 58
**SC**	–	–	**SC**	201 ± 50	208 ± 58
**Induced activity**			**Induced activity**		
**NC**	–	–	**NC**	212 ± 82	214 ± 76
**CC**	–	–	**CC**	212 ± 67	208 ± 72
**SC**	222 ± 29	210 ± 37	**SC**	224 ± 80	222 ± 63
**Amplitude** (mean ± SD)					
**Expectancy interval**			**Target interval**		
**Evoked activity**			**Evoked activity**		
**NC**	− 0.11 ± 0.03	− 0.01 ± 0.04	**NC**	0.06 ± 0.10	0.06 ± 0.11
**CC**	− 0.01 ± 0.05	− 0.02 ± 0.06	**CC**	0.06 ± 0.13	0.09 ± 0.08
**SC**	0.06 ± 0.08	− 0.01 ± 0.06	**SC**	0.01 ± 0.07	0.03 ± 0.07
**Induced activity**			**Induced activity**		
**NC**	0.01 ± 0.28	0.01 ± 0.27	**NC**	0.45 ± 0.30	0.29 ± 0.36
**CC**	0.01 ± 0.20	− 0.03 ± 0.24	**CC**	0.50 ± 0.30	0.24 ± 0.40
**SC**	0.40 ± 0.27	0.24 ± 0.22	**SC**	0.16 ± 0.19	0.07 ± 0.21

MS: multiple sclerosis; HC: healthy controls; SD: standard deviation; NC: no cue; CC: central cue; SC: spatial cue.

With regard to the induced activity, peak latency analysis of the SC conditions showed no differences between groups (Table 2).In the case of the amplitude analysis, the cue factor was statistically significant (F(2,100) = 53.89, *p* < 0.001, ƞ^2^: 0.666). However, a post hoc comparison showed no significant differences between the levels of this main factor. Nevertheless, a post hoc comparison for the following interaction “cue × anterior–posterior location and medial–lateral location” showed that the SC was higher in amplitude than theNC and CC conditions for all derivations (*p* < 0.001 in all cases) (Fig. 1 and and supplementary Table 2 for amplitude values).

Group factor also showed a significant result (F(1,50) = 4.51, *p* < 0.038, ƞ^2^: 0.136) that was caused by a higher amplitude of the gamma modulation in the MS group than in the HC group (Fig. 1 and Table 2). No significant differences were found for the group factor in terms of its interaction with location factors.

#### Target interval

In contrast to the almost nonexistent response for the cue, the evoked gamma activity for target stimuli was mainly observed for the NC and CC conditions. The latency variable was not different between cue conditions; however, the amplitude exhibited a statistically significant result for the cue factor (F(2,100) = 6.42, *p* = 0.002, ƞ^2^: 0.204) (Fig. 1 and Table 2). In a post hoc analysis, no differences were found between the levels of this main factor; however, a post hoc comparison for the interaction “cue × anterior–posterior × lateral-medial” factors revealed that all derivations exhibited a lower amplitude for the SC than for the NC or CC conditions. No significant differences were found between the NC and CC conditions (Fig. 1). Neither group nor an interaction of this factor with location factors resulted in a statistically significant difference.

With regard to the induced activity, latency did not show differences for cue or group factors. For the amplitude variable, ANOVA showed that the “cue × anterior–posterior × lateral-medial” interaction was statistically significant (F(24,1200) = 1.80, *p* < 0.009, ƞ^2^: 0.068). Post hoc comparisons showed that all derivations were higher in amplitude for the NC and CC conditions than for the SC condition (*p* < 0.001 in all cases).

Group factor also showed a statistically significant result (F(1,50) = 6.24, *p* = 0.015, ƞ^2^: 0.177), with MS patients exhibiting a higher amplitude than the healthy group (Fig. 1 and Table 2). No significant differences were found for the group factor in its interaction with other location factors.

### Low (8–10.5 Hz) and upper (10.5–13 Hz) alpha bands

#### Expectancy interval

**Figure 2:**
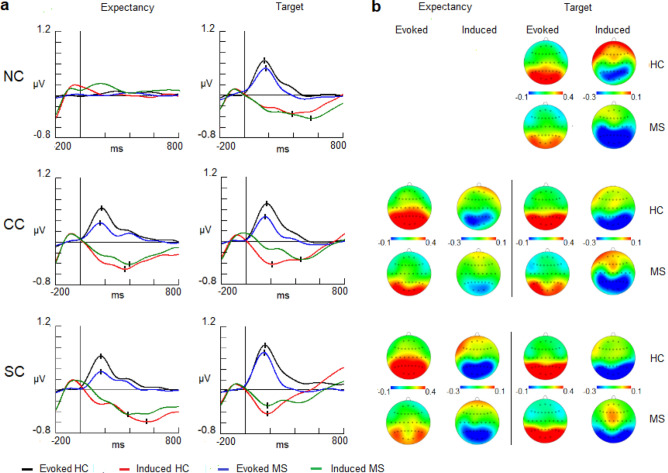
(**a**) Evoked and induced waves for the low alpha band (8–10.5 Hz) in the expectancy and target response intervals. (**b**) 2-D head maps for the peak and valley latencies of evoked and induced activity.Abbreviations: MS: multiple sclerosis; HC: healthy controls; NC: no cue; CC: Central cue; SC: spatial cue; ms: milliseconds.

**Figure 3:**
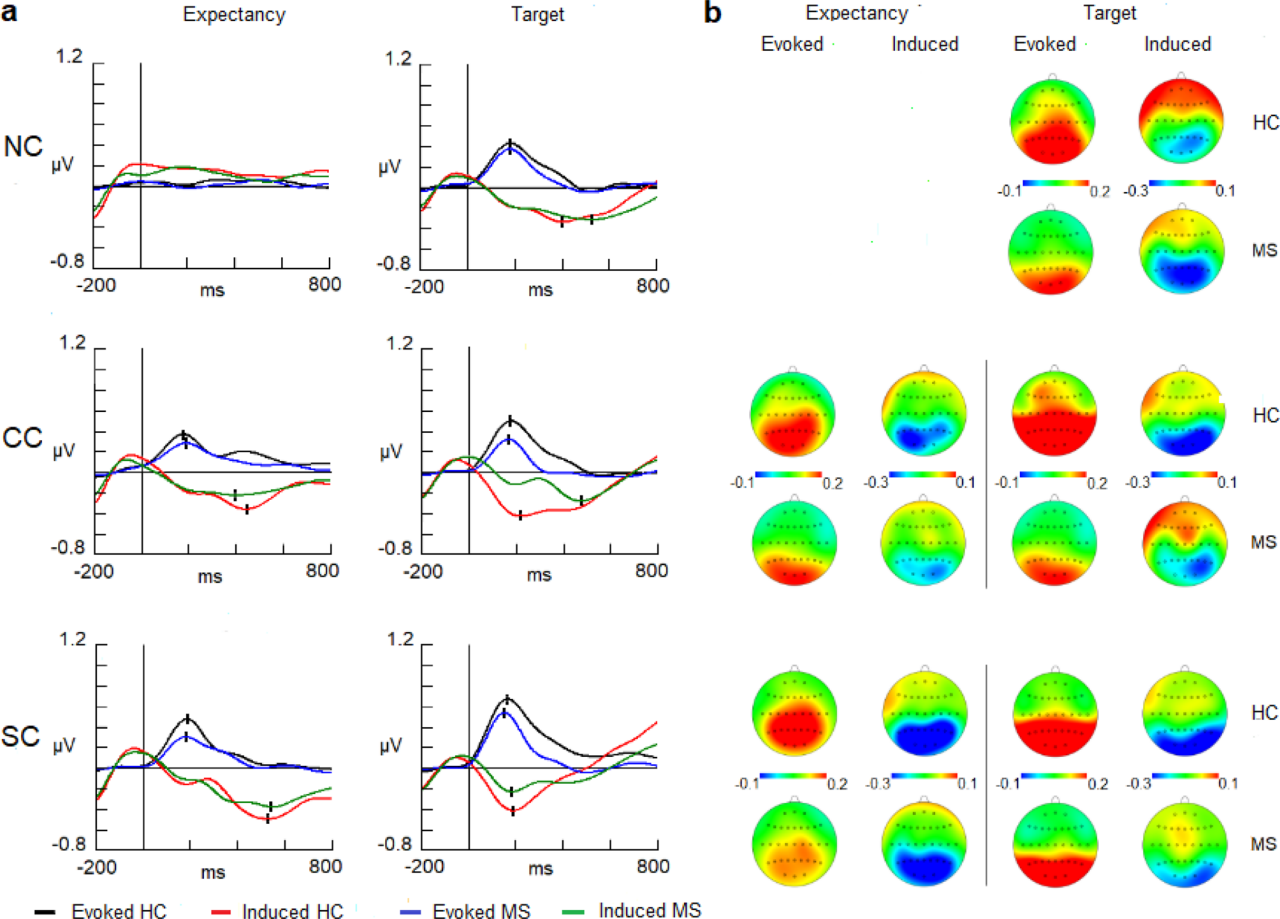
(**a**) Evoked and induced waves for the upper alpha band (10.5–13 Hz) in the expectancy and target response intervals. (**b**) 2-D head maps for the peak and valley latencies of evoked and induced activity. Abbreviations: MS: multiple sclerosis; HC: healthy controls; NC: no cue; CC: central cue; SC: spatial cue; ms: milliseconds.

In the evoked activity latency analysis, no significant differences between groups were present either in the low or upper alpha bands (Fig. 2, 3). For the amplitude variable, HCs showed larger values than patients in both the low alpha [F (1,50) = 7.1205; *p* = 0.010; η^2^ = 0.237] and upper alpha bands [F (1,50) = 4.4794; *p* = 0.039; η^2^ = 0.164] (Figs. 2, 3) (Tables 3, 4 for amplitude and latency values). Despite the differences in the amplitude in both bands, there were no differences in their topographical distributions between groups (interactions for group and location factors).

**Table 3:** Evoked and induced values for low alpha (8–10.5 Hz) and upper alpha bands (10.5–13 Hz) in the expectancy interval.

Latency (mean ± SD)					
Low alpha (8–10.5 Hz)			Upper alpha (10.5–13 Hz)		
Conditions	MS	HC	Conditions	MS	HC
**Evoked activity**			**Evoked activity**		
**CC**	173 ± 67	176 ± 45	**CC**	192 ± 78	164 ± 56
**SC**	183 ± 65	157 ± 42	**SC**	173 ± 70	187 ± 62
**Induced activity**			**Induced activity**		
**CC**	403 ± 96	362 ± 158	**CC**	370 ± 80	441 ± 90
**SC**	542 ± 77	556 ± 68	**SC**	556 ± 60	519 ± 75
**Amplitude** (mean ± SD)					
**Low alpha (8**–**10.5 Hz)**			**Upper alpha (10.5**–**13 Hz)**		
**Evoked activity (0**–**350 ms)**			**Evoked activity (0**–**350 ms)**		
**NC**	0.004 ± 0.14	0.023 ± 0.12	**NC**	0.001 ± 0.09	0.015 ± 0.08
**CC**	0.155 ± 0.22	0.259 ± 0.23	**CC**	0.093 ± 0.14	0.160 ± 0.17
**SC**	0.181 ± 0.22	0.277 ± 0.23	**SC**	0.131 ± 0.16	0.181 ± 0.17
**Induced activity (0**–**350 ms)**			**Induced activity (0**–**350 ms)**		
**NC**	0.103 ± 0.21	0.061 ± 0.23	**NC**	0.057 ± 0.15	0.084 ± 0.22
**CC**	− 0.098 ± 0.24	− 0.145 ± 0.20	**CC**	− 0.077 ± 0.17	− 0.110 ± 0.19
**SC**	− 0.101 ± 0.19	− 0.129 ± 0.22	**SC**	− 0.087 ± 0.14	− 0.101 ± 0.20
**Induced activity (350**–**700 ms)**			**Induced activity (350–700 ms)**		
**NC**	0.053 ± 0.23	0.034 ± 0.22	**NC**	0.029 ± 0.16	0.067 ± 0.24
**CC**	− 0.190 ± 0.34	− 0.248 ± 0.36	**CC**	− 0.121 ± 0.28	− 0.168 ± 0.26
**SC**	− 0.260 ± 0.48	− 0.277 ± 0.43	**SC**	− 0.204 ± 0.38	− 0.207 ± 0.31

MS: multiple sclerosis; HC: healthy controls; SD: standard deviation. NC: no cue; CC: central cue; SC: spatial cue.

**Table 4:** Evoked and induced values for low alpha (8–10.5 Hz) and upper alpha bands (10.5–13 Hz) in the target interval.

Latency (mean ± SD)					
Low alpha (8–10.5 Hz)			Upper alpha (10.5–13 Hz)		
Conditions	MS	HC	Conditions	MS	HC
Evoked activity			Evoked activity		
**NC**	170 ± 55	167 ± 48	**NC**	172 ± 49	174 ± 66
**CC**	158 ± 57	172 ± 49	**CC**	167 ± 71	170 ± 63
**SC**	154 ± 52	158 ± 38	**SC**	155 ± 38	156 ± 44
**Induced activity**			**Induced activity**		
**NC**	540 ± 81	404 ± 78	**NC**	487 ± 82	414 ± 76
**CC**	473 ± 72	195 ± 73	**CC**	466 ± 67	206 ± 72
**SC**	191 ± 80	178 ± 55	**SC**	183 ± 80	180 ± 63
**Amplitude** (mean ± SD)					
**Low alpha (8**–**10.5 Hz)**			**Upper alpha (10.5**–**13 Hz)**		
**Evoked activity (0**–**350 ms)**			**Evoked activity (0**–**350 ms)**		
**NC**	0.070 ± 0.36	0.353 ± 0.35	**NC**	0.141 ± 0.09	0.216 ± 0.08
**CC**	0.277 ± 0.31	0.410 ± 0.32	**CC**	0.134 ± 0.15	0.235 ± 0.17
**SC**	0.376 ± 0.34	0.476 ± 0.33	**SC**	0.218 ± 0.16	0.329 ± 0.17
**Induced activity (0**–**350 ms)**			**Induced activity (0**–**350 ms)**		
**NC**	− 0.183 ± 0.35	− 0.148 ± 0.26	**NC**	− 0.106 ± 0.15	− 0.129 ± 0.22
**CC**	− 0.106 ± 0.29	− 0.257 ± 0.33	**CC**	− 0.071 ± 0.17	− 0.220 ± 0.19
**SC**	− 0.177 ± 0.25	− 0.261 ± 0.23	**SC**	− 0.128 ± 0.14	− 0.207 ± 0.20
**Induced activity (350**–**700 ms)**			**Induced activity (350**–**700 ms)**		
**NC**	− 0.360 ± 0.67	− 0.262 ± 0.56	**NC**	− 0.270 ± 0.16	− 0.205 ± 0.24
**CC**	− 0.300 ± 0.54	− 0.215 ± 0.57	**CC**	− 0.203 ± 0.27	− 0.150 ± 0.26
**SC**	− 0.208 ± 0.36	− 0.056 ± 0.54	**SC**	− 0.099 ± 0.38	0.037 ± 0.31

MS: multiple sclerosis; HC: healthy controls; SD: standard deviation. NC: no cue; CC: central cue; SC: spatial cue.

Regarding induced activity, the latency in which the maximum amplitude was reached was different for each cue condition (Figs. 2, 3). This difference for the cue factor was observed both in the low alpha [F (1.50) = 0.77980; *p* < 0.001; η^2^ = 0.714] and upper alpha bands [F (1.50) = 6.3649; *p* = 0.014; η^2^ = 0.773]. A post-hoc Bonferroni comparison showed that the SC condition reached the maximum amplitude later than the CC condition (in both the low and upper alpha bands, *p* < 0.001) (Tables 3, 4 for latency values).

Despite the larger amplitude observed in the control group (Figs. 2, 3), no statistically significant differences for group factor were observed throughout the expectancy interval divided into two time intervals (0–350 ms and 350–700 ms) or in its interaction with other location factors (Tables 3, 4 for mean amplitude values).

#### Target interval

Evoked latency showed no significant differences between the two groups in the low alpha and upper alpha subbands (Figs. 2, 3). HCs showed a larger amplitude than MS patients in both the low alpha [F (1.50) = 8.4645; *p* = 0.005; η^2^ = 0.242] and upper alpha bands [F (1.50) = 4.5503; *p* = 0.037; η^2^ = 0.125] (Figs. 2, 3 and Tables 3, 4 for amplitude and latency values). There were no differences in topographical distribution between groups.

In the induced latency analysis, the interaction of group and cue factors was significant in both the low and upper alpha bands [F (2.204) = 81.212; *p* < 0.001; η^2^ = 0.599] [F (2.204) = 87.305; *p* < 0.001; η^2^ = 0.644] (Figs. 2, 3).A post-hoc Bonferroni comparison showed that MS patients in the NC and CC conditions reached the maximum amplitude later than controls in both alpha subbands (*p* < 0.001). However, in the SC condition, there were no differences between groups in the latency in the low alpha or upper alpha bands (Figs. 2, 3).

With regard to the amplitude variable for induced modulation, no differences in topographical distribution were found for the group factor or its interaction with other location factors in either low alpha or upper alpha bands or in any time interval analyzed (0–350 ms and 350–700 ms) (Figs. 2, 3 and Tables 3, 4 for mean amplitude and latency values).

#### Phase analysis for induced and evoked activity

**Figure 4:**
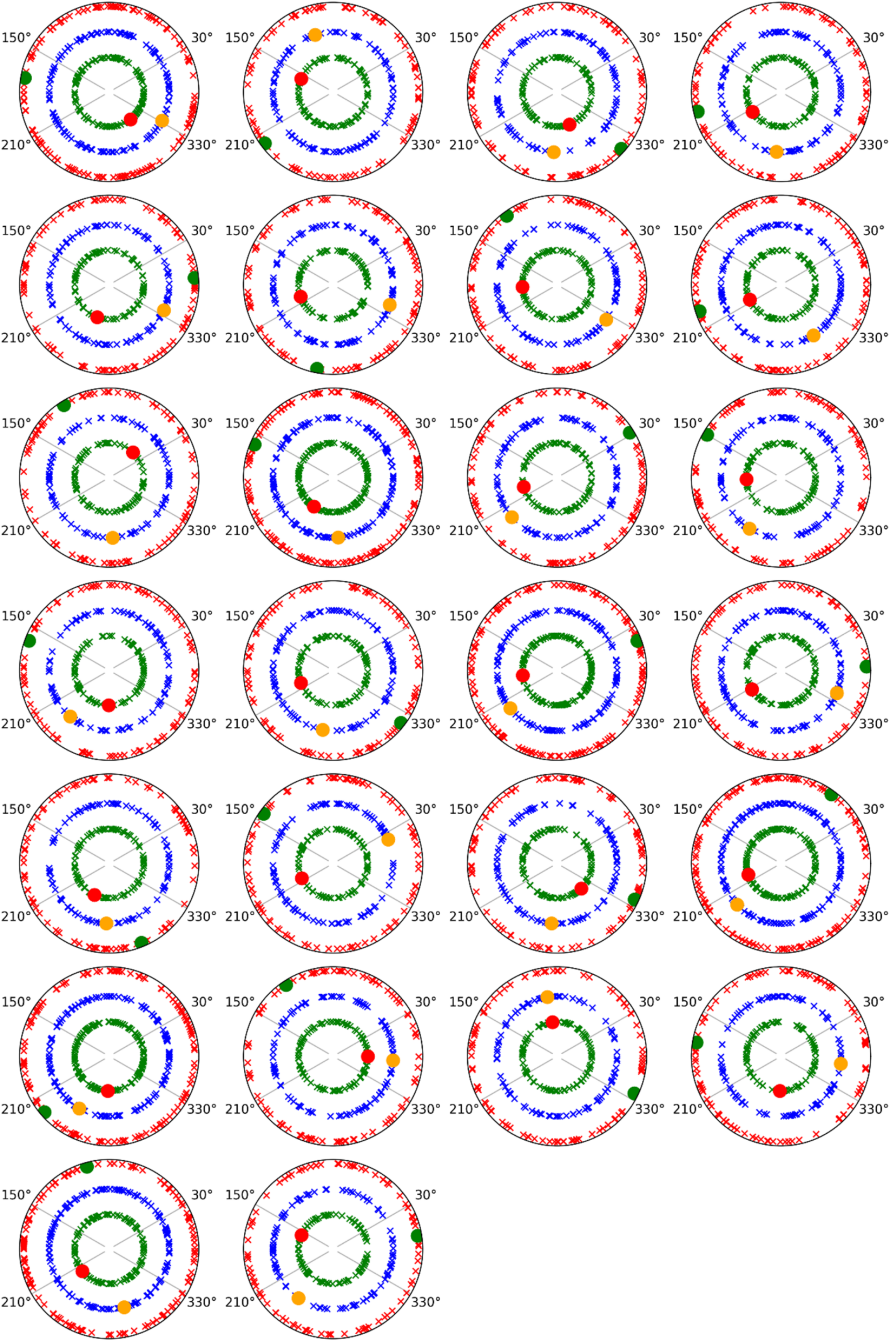
Plotting of phase values in the lower and upper alpha and gamma bands for evoked and induced activities and in the target response interval following the presentation of a central cue and for each healthy control subject (26 subjects). Evoked activity is represented by red, yellow and green dots, and induced activity is displayed with green, blue and red crosses for lower alpha (8–10.5 Hz), upper alpha (10.5–13 Hz) and gamma (30–45 Hz) bands, respectively.

After phase analyses were made for the evoked and induced activities in the frequency bands (low and upper alpha and gamma bands) and for both groups, it was possible to conclude that the potential contribution of the evoked activity over induced activity could be discarded in all bands, as has been described in previous studies^52,55^. Figure 4 shows that the phase values of induced activity for HCs in the lower and upper alpha subbands and gamma band for the target stimuli following a central cue showed random values that were not concentrated in the phase value of the evoked activity. Similar results were observed in other cue conditions and for the MS group (see Figs. 1–7 in supplementary material).

## Discussion

The behavioral results suggested an attentional impairment in the MS group. A general slowing was observed (approximately 100 ms) for MS patients in all cue conditions. Moreover, the delay in reaction times was not caused by a speed-accuracy tradeoff between experimental conditions for the patient group compared with the HC group. In addition, the neuropsychological assessment revealed an attentional impairment that was not caused by severe signs of depression (low scores on the BDI).

A delay in the reaction time for attentional tasks in MS patients has been described in previous studies^18,56–58^. With regard to the neural networks, the slower reaction times in the NC and CC conditions suggest that alerting mechanisms (phasic or tonic) could be impaired, as has been suggested by other studies^16–18^. Moreover, a longer reaction time in the SC condition also indicates that the orienting mechanism could also be affected, as previously described^18^.

However, it seems unreasonable that such a common delay in all cue conditions (100 ms) could be due to multiple and diverse impairments such as the phasic and tonic alerting or orientation mechanisms. It is not possible with the current results to discard that a common mechanism operating in all conditions could be the reason for the general slowing in the MS sample.

A relevant result to better understand the attention impairment in the MS group comes from the data after the correction of Fernandez-Duque and Black^53^. A statistical *t* test group comparison in all cue conditions (e.g., NC MS vs NC HC) showed that the NC condition was responded to faster in the internal comparisons for MS patients than the same comparison in the HC group. This result suggests that MS patients try to be more ready for uncued targets that are more demanding in terms of response time. This approach could be a strategic compensation from MS patients to reduce the average reaction time for all the experimental conditions. However, a more intricate interpretation is suggested from neurophysiological measures, which will be described in the following paragraphs.

Regarding the induced activity, the gamma band seems to be related to the translation of the attentional focus towards the cued visual spatial locations. The only condition in the expectancy interval that showed an increase in the gamma amplitude was the SC condition during which subjects were required to move their attentional focus to specific spatial regions indicated by cues (100% valid). On the other hand, during the NC or CC conditions, the attentional focus is maintained around a fixation point; therefore, no increase in the gamma activity is observed.

In the case of the induced gamma response for target stimuli, the NC and CC conditions need an orienting response provoked by the onset of the stimuli and consequently a shifting of the attention focus towards it. This shift is translated as an increase in the amplitude of the gamma activity as it occurred in the SC condition for the cue onset. In the case of the target preceded by the SC condition, a small increase in the gamma response could represent a lower need to readjust the attentional focus precisely over the central arrow to avoid competition from distractor stimuli.

These results are in accordance with those of a previous study that showed that the orientation mechanism modulates gamma activity (without a specific definition of evoked or induced activity) in the first 200 ms after the onset of the target^47^. Gamma band activity has been associated with an attentional mechanism involved in optimal performance in cognitive tasks^37, 39^.

With regard to the group variable, a first consideration is that the peak latencies of the evoked or induced gamma activities were not different between both groups. It is well known that MS pathology usually implies delays in the timing of cognitive processes caused by the demyelination process^59–61^. However, there have been diverse studies that have not found latency delays in behavioral or EEG parameters in MS patients during the execution of cognitive tasks^20,62^.

At the same time, the amplitude for induced gamma activity was higher for MS subjects than for the HC group. It is counterintuitive that the MS group exhibited a higher amplitude than and a similar latency to the HC group. However, if the functional meaning of the induced gamma modulation represents the translation of the attentional focus in the visual field, a possible interpretation is that the ability of the patients to adequately keep this focus in an appropriate location (e.g., on the fixation point) is impaired, and constant reorienting processes are needed with a consequent increase in the gamma amplitude. In this case, the gamma band reflects a compensatory mechanism rather than an impaired one.

A corollary of this interpretation is that MS patients probably need to increase their engagement of alerting and orienting mechanisms to improve their reaction in each trial to relocate their attentional focus as fast as possible. The maintenance of this strategy to compensate for the deficit is probably the cause of cognitive fatigue observed in some cases among MS patients during sustained attention tasks^63,64^.

Finally, it is necessary to comment about the difference between evoked and induced gamma activity. Notably, evoked gamma modulation was mainly present for the target stimuli, and induced activity was linked to both stimuli (cue and target). It could be suggested that evoked activity is only related to the relevant stimuli that require a motor response; in contrast, induced activity does not depend on that feature. Unfortunately, the present experiment does not allow further assessment of this issue, and future studies are necessary to disentangle the precise functional role of both gamma modulations in this respect.

With regard to evoked activity that may mainly represent the alpha content of the early ERPs (P1 and N1), there was no difference between groups for the latency parameter. Therefore, it could be suggested that the described slowing in the latency of early ERPsin MS patients^18,58,65,66^ due to the demyelination was not observed in our data. However, other studies did not find differences in early visual ERP latency between HCs and patients with MS^67^.

On the other hand, with regard to the amplitude parameter, previous studies have found lower amplitudes in the P1 component^68^ and N1-P2 complex^69,70^among MS patients. Our results are in agreement with these studies in terms of both the cue and target early evoked responses. This decrement in the amplitude could reflect that a lower number of neurons were synchronized to elicit the evoked response but at the same time preserved their connectivity, which maintained the appropriate latency. Other authors have interpreted similar results as an impaired mechanism of synchrony in the neural generators of the early visual processing in MS group^71^.

With regard to the induced alpha activity, a clear negative trend was observed in the CC and SC conditions in the expectancy interval. It has been suggested that the onset of alpha desynchronization starting at approximately 300 ms is a sign that the influence of the alpha band on cortical activity was disappearing and that other task-relevant processes/bands took place^25^. This reduction of alpha synchronization observed in our study could also be in accordance with the findings of previous studies in which a decreased alpha band was linked to anticipatory attention (orienting network) with respect to the arrival of the target in a cue paradigm^26^.

In the particular case of alpha modulations during the ANT, a study found a decrease in alpha activity (not distinguished between phase or nonphase) related to the alerting network from 200 to 450 ms after the onset of the cue^47^. These authors indicated that this desynchronization could be related to the abrupt onset of the cue that engages an alerting response. However, in our study, the cue factor results from ANOVA indicated that the CC condition reached its highest value before the SC condition. We suggest that the reason for this difference may be the different information content involved in the two cues. The CC condition provides timing information, while the SC condition adds spatial information to the timing expectancy. The more information is added, the more latency is necessary to reach the maximum desynchronization. Previous studies have demonstrated in the time domain of EEG [contingent negative variation (CNV)] that the more spatial and timing information was given in the cue, the higher the amplitude of the CNV^72^. However, the CNV trend is maintained until the target stimuli are presented (at least in a one-second SOA), but induced alpha modulation reaches maximum negativity and starts recovering to the baseline level before the target appears. This last modulation seems to be more related to a specific mechanism in a determined time interval than to a sustained preparatory state for incoming stimuli.

This difference between the CC and SC conditions observed in both alpha bands (low and upper) could indicate that both general (alerting) and specific (orienting) attentional mechanisms are recruited in the expectancy interval. In particular, some authors have suggested that the decrease in the upper alpha band may be related to anticipatory attention^26^or maintaining attentional focus^73^.

With regard to the comparison between both experimental groups, no differences were found in the latency or amplitude. These results suggest that expectancy mechanisms indexed by alpha-induced activity seem preserved in MS patients. A previous study in our laboratory with the same cognitive task demonstrates that the expectancy mechanisms related to the CNV showed alterations in MS patients^18^, which supports that evoked activity and induced activities, can be disentangled and represent different cognitive mechanisms. Moreover, phase analyses showed that single trial-induced phases displayed random values that were not concentrated in the evoked phase values (Fig. 4 and supplementary material Fig. 1). These results suggest that it is possible to discard the potential contribution of the evoked activity over the induced activity in terms of amplitude modulation. Therefore, the induced activity is truly non-phase-locked activity and is not caused by the jittering of the evoked activity. A similar result was obtained in our laboratory during the performance of an oddball task^52^.

In the target response interval, alpha-evoked activity showed no differences in the latencies of the responses for the target in all cue conditions (NC, CC and SC). On the other hand, the amplitude showed a reduction in the MS group suggesting an impaired processing of the target stimuli. Some studies have reported that the early components (P1/N1) in a cueing paradigm are enhanced for the target stimuli as the result of sensory preparation^74–76^. However, in the present study, it is not possible to determine if the decrement was caused by an impaired preparatory mechanisms in MS patients (not shown in the current experiment but demonstrated in previous studies^18^) or was due to a reduced number of neurons operating as it was suggested for the cue processing. Further research is needed to clarify the precise factors involved in the decrement of early evoked activity in MS pathology.

With regard to the induced activity, different modulations were found for target stimuli depending on the preceding cue. In the NC and CC conditions, there was no difference in the amplitude between experimental groups. However, an increase in the latency of the maximum negativity for both cue conditions was observed in the MS group. It seems that patients performed a first alpha desynchronization in the first valley (0–350 ms) but not well enough in terms of efficient cognitive processing (lower amplitude) and rebounded with a second valley that reached a better level of alpha desynchronization.

Moreover, the SC condition did not show a statistically significant delay for MS patients, with a slightly higher amplitude in the first than in the second valley. However, the presence of a second valley can be observed in the SC condition for MS patients compared with controls, suggesting that a rebound could be present in some trials. These rebounds in the decrease in the alpha modulation could reflect an effort from MS patients to compensate for cognitive impairment, as has been suggested in previous studies^43^.

In summary, the current behavioral results supported previous studies^16–18^ that suggested an impairment in the alerting and orienting networks in MS patients. However, diverse neurophysiological parameters suggest a more complex interpretation of the causes for cognitive impairment in this MS sample.

Our hypothesis after the analysis of the current experiment is that MS patients exhibited an impairment to maintain attentional focus during the experiment, as has been suggested in previous studies^73^. First, this hypothesis could explain why the evoked activity for cue and target stimuli is reduced considering that MS patients are not placing their attentional focus precisely in the relevant locations for the experiment. Second, an increased induced gamma modulation in MS patients could reflect a higher need of reorienting in both cue and target presentations. However, the latency of the gamma peak was not delayed in the MS group, which suggests that the relocation of the spatial attentional focus is not enough to justify the delay in behavioral responses.

This last evidence leads us to consider that the impairment in the maintenance of attentional focus not only involves its spatial properties but also involves its object properties. Multiple studies have analyzed the interaction between both determinants of the stimuli (spatial and object features) and described its neural sources^77–79^. Our conclusion is that MS patients have difficulties in maintaining an appropriate “spatial-object” attentional focus. As it has been pointed out in a study with ADHD children^80^, an impaired attentional performance is not caused necessarily by the orienting process itself but by the difficulty in maintaining the relevant information demanded for the task (spatial and object properties) during the preparation interval and until the target processing stage. The maintenance of an attentional focus to perform the ANT could also be related to working memory mechanisms^81–83^. Considering this link between the two processes, diverse studies have described working memory impairments in cognitive tasks in patients with MS^83–85^**.**

The correlate for the difficulty in the processing of object properties of the target could be represented by the rebounds in the induced alpha modulation. However, this impaired mechanism is a consequence of the poor maintenance of the spatial-object attentional focus in MS patients. The precise neurophysiological parameter for the specific impairment in the maintenance of the spatial-object attentional focus has not been found in the present experiment and future studies are needed for its identification.

In any case, the study of the nonphase activity in EEG signals has exhibited hidden cognitive mechanisms (compensatory and impaired) for the traditional “evoked studies” that can complete our knowledge of the neural basis of cognitive impairment in MS patients and other pathologies.

## Methods

### Participants

Twenty-six patients with a relapsing–remitting MS (RRMS) form were recruited in the Multiple Sclerosis Unit of Hospital Universitario Virgen Macarena (Seville, Spain). The definitive diagnosis was made by a neurologist, according to McDonald's criteria^85^. To be eligible for inclusion in the study, patients were required to be under an EDSS of 6 (mean 2.4, SD 1.5) and avoid exclusion conditions: clinical relapses in the last month; presence of comorbid neurodegenerative or psychiatric disorders; severe signs of depression; history of substance abuse; head trauma; vascular diseases and seizures; significant upper limb impairment; or visual acuity or field deficits.

The MS sample was formed by 16 women and 10 men, with an age range of 29–45 years (mean 34.42, SD 7.07); one of the patients was left-handed. An HC group was selected with sociodemographic variables matched to those off the MS group (11 women and 15 men who were between the ages of 22–52 years (mean 30.30, SD 9.30); one of the HCs was left-handed. All the healthy subjects were declared to be free of neurological conditions.

This study was carried out in compliance with the Helsinki Declaration. All participants signed informed consent before their inclusion, and the protocol was approved by the Ethics Committee of the University of Seville (project code: PSI2016-78,133-P).

### Neuropsychological and depression assessments

Neuropsychological and depression assessments of the patients were performed by well-trained psychologists blinded to the study goals. The attention and speed of information processing were evaluated through the Paced Auditory Serial Addition Test, 3s (PASAT-3s)^86,87^ and the Symbol Digit Modality Test (SDMT)^88,89^. Cognitive impairment was identified using the normative scores developed by Sepulcre^54^. The Beck Depression Inventory (BDI-II)^90,91^ was used to assess symptoms of depression.

### Cognitive task

**Figure 5:**
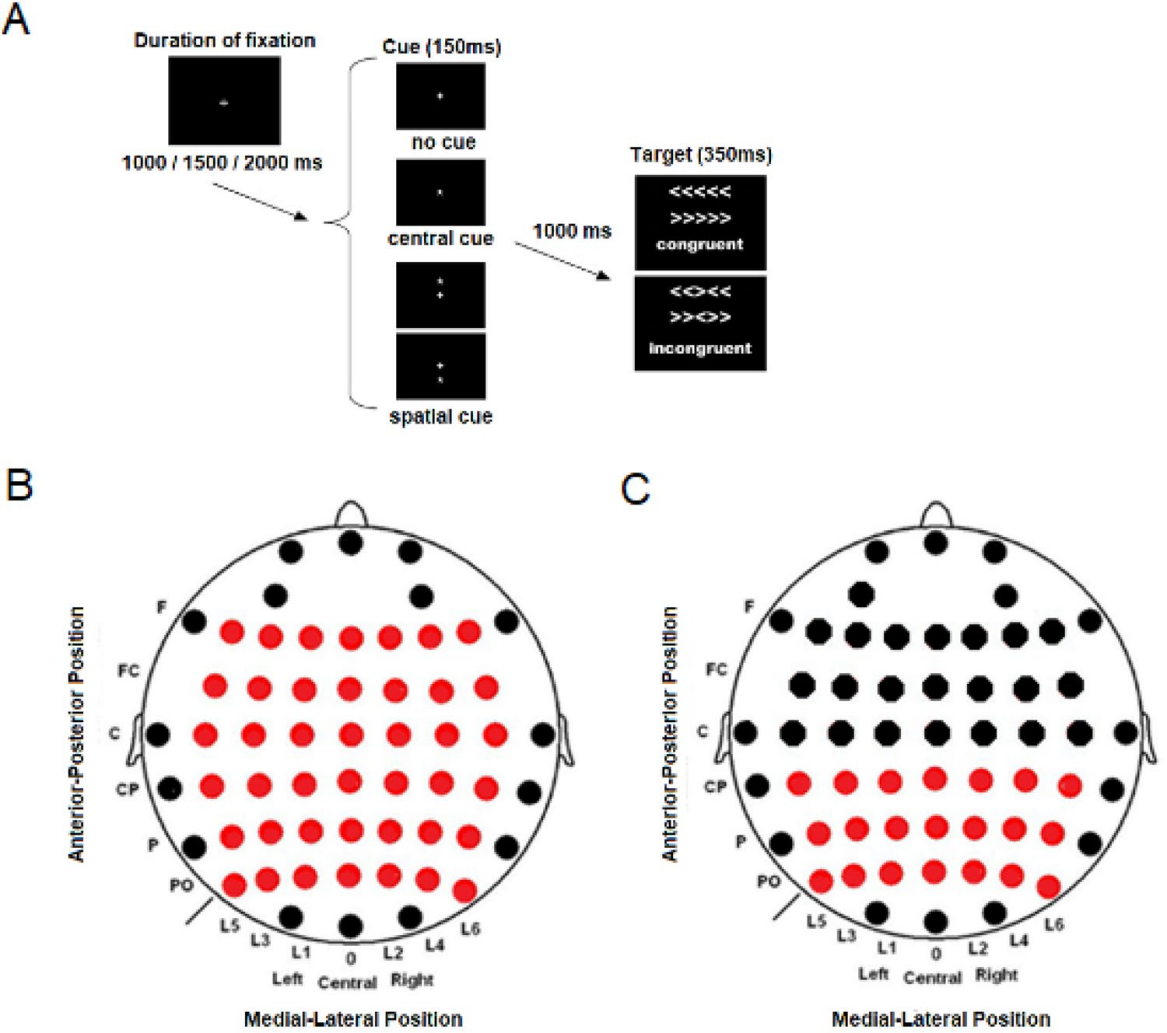
(**A**) Schematics of the Attention Network Test (ANT). (**B**) 6 × 7 electrode matrix. (**C**) 3 × 7 electrode matrix.

Participants were seated in a sound-attenuated room in front of a computer monitor. Stimuli were created by E-prime 2.0 (Psychology Software Tools, Inc. Pittsburgh, PA) and presented on an LCD screen. An adaptation of the ANT was used for this study^11^ (Fig. 5A).Both cue and target stimuli were presented on a black background on which a fixation cross was projected in the middle of the screen throughout the experiment. The task consisted of two intervals (defined by the onset of the cue and target), and the subject only had to respond to the target stimuli. During the expectancy interval (between the cue and target), there were 3 possible cue conditions. The no cue condition (NC) consisted only of the presentation of the fixation cross in the middle of the screen and a delayed presentation of the target stimulus. The central cue (CC) condition involved an asterisk (cue) being presented in the middle of the screen and providing the subject with a temporal clue about the timing (because of a fixed SOA, 1 s) of the onset of the target. The last condition was the spatial cue (SC) condition, in which the asterisk appeared above or below the fixation cross, providing both clues about the timing of the target stimulus presentation and spatial location (cues were 100% valid). All cue conditions appeared in the same proportion. The cues were presented with a visual angle of 0.41 on the × axis and 0.41 on the Y axis.

In the target response interval, the participant had to respond to stimuli formed by 5 arrows pointing to the left or right side. The presented stimuli could be congruent (C) when the central arrow (target) was oriented in the same direction as the flankers or incongruent (I) when the flanker arrows pointed to the opposite direction of the central arrow. The target stimulus was presented at a total visual angle of 3.28 on the X-axis and 0.41 on the Y-axis and 0.86 above or below the fixation cross. Congruent and incongruent stimuli were presented in equal proportions. Furthermore, half of the targets pointed to the right and half to the left. The subject was required to respond according to the direction of the central arrow by pressing the left button with the left thumb when the central arrow pointed to the left or by pressing the right button with the right thumb when the central arrow pointed to the right.

The timing for the presentation of stimuli was modified from the original ones^11^ to adapt it for MS patients. The cue duration was 150 ms, and the target stimulation presentation lasted 350 ms. The time between the end of the cue and onset of the target was 1000 ms. Participants had 1000 ms to respond from the onset of the target stimulus. The duration between the end of one trial and the next one was variable (1000, 1500 or 2000 ms). The experiment was divided into two blocks of 144 tests each, for 288 in total. A pause between blocks was proportionate to avoid fatigue in participants. The duration of this pause was defined by the need of the participant but usually did not last for more than two minutes. The total duration of the experimental procedure with an average pause block of two minutes was approximately of 15 min. The presentation of the trials (cue and target pairs) was set in a pseudorandom order.

Reaction times and precision percentages were calculated for all conditions. All participants were instructed to respond as quickly and accurately as possible. To analyze differences between groups in specific conditions, a potential general slowing for reaction time in MS patients was corrected following the recommendations of Fernandez-Duque and Black^53^. No network effects were calculated because diverse studies have concluded that thesevalues can induce erroneous interpretations of the data^72,92^.

### EEG recording and analyses

EEG data were recorded from 58 electrodes (Ag/AgCl) in standard locations of a 10–10 system^93^and amplified with BrainAmp amplifiers (Brain Products GmbH, Germany) (Fig. 5B, 5C for detailed positions of electrodes). The EEG signal was filtered online with a bandpass of 0.01 to 100 Hz, digitized with a sampling rate of 500 Hz and stored using Brain Vision Recorder software (Brain Products GmbH, Germany). These continuous data were referenced online to the linked auricular lobes and offline to a common averaged reference. Impedance was kept below 5 kOhm during the experiment. Vertical and horizontal electro-oculograms (VEOG and HEOG) were also recorded with a bipolar montage. Trials with an HEOG signal outside the ± 50 µV range were rejected. For blinking artifacts, ocular correction was performed in the scalp electrodes using the algorithm developed by Gratton et al.^94^. A continuous signal was epoched in segments of − 200 to 1000 ms, with zero being the onset of the cue or target stimuli. A baseline correction (− 100 to 0 ms) was also applied to all conditions.

**Figure 6:**
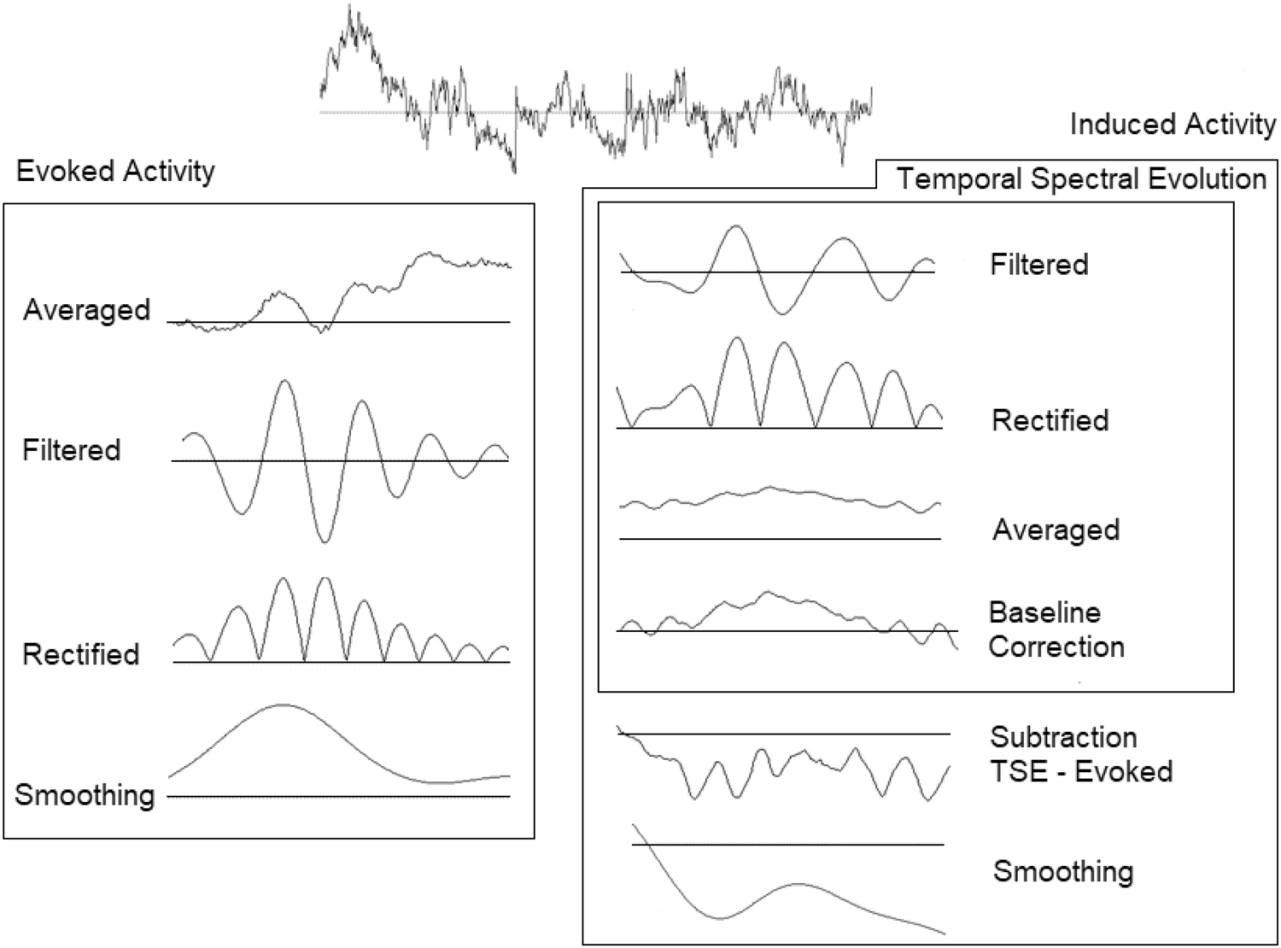
Schematics of EEG signal processing used to obtain evoked and induced activities.

After all this processing, two possible analyses were made for the EEG signal: to obtain evoked activity, all conditions (NC, CC and SC, for both cue and target stimuli) were averaged independently, filtered for low (8–10.5 Hz) and upper (10.5–13 Hz) alpha and gamma bands (30–45 Hz) (in all cases, 48 dB/octave, Butterworth) and rectified^52^.In the case of the induced activity, a temporal spectral evolution (TSE) was calculated with the following steps: identical bandpass filtering in previously defined low and upper alpha and gamma bands performed over EEG epochs, rectifying the signal and finally averaging for all stimuli conditions. After this analysis, a subtraction of evoked activity from the TSE was subsequently performed to calculate the induced response (nonphase activity)^51^ (see Fig. 6).

To analyze the latency of all band modulations as suggested by Keil^95^, latency was defined at the electrode with the maximum amplitude observed in a grand average of all experimental conditions and performed for each group. Following these criteria, the evoked gamma activity in the expectancy interval was almost nonexistent in all cue conditions, and induced activity was mainly present in the SC condition and for the interval of 210–225 ms. In the case of the target stimuli, the peaks of the evoked and induced gamma activity occurred in the interval of 175 to 245 ms. Once the intervals were set, calculations of gamma peak latencies were made for each participant. Moreover, the mean amplitude value was exported for the entire intervals defined previously in a matrix of 3 × 7 electrodes that covered the posterior area of the scalp where gamma activity was prominent (Fig. 5C).

With respect to the low and upper alpha subbands, the evoked activity was analyzed in both expectancy and target response intervals for the 0–350-ms interval. The latency peak was determined in this interval individually for each participant. Amplitude analyses were performed with mean values in the 0–350 ms and for a matrix of 6 × 7 electrodes in the expectancy interval and a 3 × 7 matrix in the target response interval.

With regard to the induced activity, the valley latency value was identified as the maximum negativity present in any of the intervals analyzed (0–350 ms; 350–700 ms). Individual values were calculated for each subject of both experimental groups. Amplitude studies were performed as for the evoked activity but in two different time intervals (0–350 ms; 350–700 ms).

### Phase analysis for evoked and induced activity

Because of a potential contribution of evoked over induced activity could occur (especially in the target response), the induced modulation was checked to ensure that it was truly nonphase locked activity for all bands studied (low/upper alpha and gamma bands). To achieve this goal, the evoked response was estimated through averaging over trials and then subtracted from each of the individual trials^55,96^. To do so, the trials were filtered in the desired bands (low alpha: 8–10.5 Hz; upper alpha: 10.5–13 Hz; gamma: 30–45 Hz, in all cases, 48 dB/octave, Butterworth), and the Hilbert transform was applied to calculate instantaneous phase.

The phases of gamma activity were measured at the interval of 210–225 ms (expectancy interval) and 175–245 ms (target response interval) in single trials, which corresponds to the estimated grand average peak interval of the induced response. In the case of the alpha bands, phase calculations were performed in 0–350 ms for low and upper alpha subbands following the same protocol described for the gamma band. Last, the phases of the evoked responses (alpha and gamma) were calculated by following the same protocol performed for the induced activities and in the same time intervals.

### Statistical analyses

#### Behavioral responses

A previous exploratory analysis of the data revealed that there were no differences by the “congruency” variable or in its interaction with the experimental group for alpha or gamma modulations. For this reason and to facilitate the following analyses, we collapsed congruent and incongruent values for each cue condition.

By means of the Shapiro–Wilk test (*p* > 0.05), all variables were checked for normality. Parametric (ANOVA) or nonparametric tests (Kruskal–Wallis) were performed to study possible differences in behavioral and neurophysiological variables where appropriate.

The analysis of the reaction times was carried out with a two-factor ANOVA: group factor (levels: HC and MS patients) and a cue factor (levels: no cue, central cue and spatial cue). After correction suggested by Fernández-Duque and Black^53^, the *t* test was performed to check whether both groups responded differently to the cues (paired comparisons for each cue condition value). The accuracy of the subjects' responses was analyzed applying the Kruskal–Wallis test with the same factors as for the reaction time ANOVA.

#### Gamma band

To analyze the latency of evoked and induced gamma activities in the expectancy interval, we performed two independents ANOVAs to compare both experimental groups only for the SC condition. For the amplitude variable, we performed two ANOVAs (evoked and induced) with the following factors and levels: group factor (levels: HC and MS patients); cue factor (levels: NC, CC and SC); anterior–posterior location factor (levels: mid-parietal, parietal and parietal-posterior); and medial–lateral location factor (levels: line 5, line 3, line 1, central, line 2, line 4, line 6).

In the target response interval, evoked and induced gamma latencies were analyzed separately by ANOVAs including all cue conditions (NC, CC and SC) and for both experimental groups. The amplitude variable was studied with the same factors as described for the expectancy interval.

#### Low and upper alpha subbands

In the case of the analysis of alpha activity (both subbands), in the expectancy interval, evoked and induced latencies were analyzed separately. The two ANOVAs performed had two main factors: “group” (levels: HC and MS patients) and “cue” (levels: CC and SC). The NC condition was not introduced because of a null response of evoked and induced activity due to the absence of cue stimulation. With regard to topographic study, the evoked amplitude was analyzed with an ANOVA with the following factors: group (levels: HC and MS patients); cue factor (levels: NC, CC and SC); anterior–posterior factor (levels: frontal, frontocentral, central, mid-parietal, parietal and parietal-posterior); and lateral-medial factor (line 5, line 3, line 1, central, line 2, line 4, line 6). In the case of induced activity, two independent ANOVAs were performed for each time interval (0–350; 350–700) with the previously described factors for evoked activity.

In the target response interval, evoked and induced latencies were analyzed separately with the same ANOVA factors as in the expectancy interval, but in this case, all the cues were included in the cue factor (levels: NC, CC and SC). On the other hand, amplitude analysis of evoked and induced modulations was performed with the same factors for the ANOVAs in the expectancy interval but with fewer levels for the anterior–posterior factor (levels: mid-parietal, parietal and parietal-posterior) (matrix 3 × 7).

In all the analyses described above, sphericity was corrected with Greenhouse–Geisser, and a statistically significant result was considered from *p* < 0.05. A post hoc analysis of all the possible comparisons was carried out using Bonferroni correction.

## Supplementary information

Supplementary information 1.

Supplementary information 2.

## Data Availability

The datasets generated during and/or analyzed during the current study are available from the corresponding author on reasonable request.
