# Supplementary material for: Altered phase and nonphase EEG activity expose impaired maintenance of a spatial-object attentional focus in multiple sclerosis patients
Source: Sci Rep. 2020 Nov 26;10:20721. doi: 10.1038/s41598-020-77690-y (PMC7691340; doi:10.1038/s41598-020-77690-y)
Supplement: Supplementary file 2 — Supplementary information 2. [file 41598_2020_77690_MOESM2_ESM.docx]

**Full title:**

Altered phase and nonphase EEG activity expose impaired maintenance of a spatial-object attentional focus in multiple sclerosis patients **Authors:**

Vazquez-Marrufo M^1*^, Sarrias-Arrabal E^1^, Martin-Clemente R^2^, Galvao-Carmona A^3^, Navarro G^4^, Izquierdo G^5^

(1) Experimental Psychology Department, Faculty of Psychology, University of Seville, Spain.

(2) Signal Processing and Communications Department, Higher Technical School of Engineering, University of Seville, Spain.

(3) Department of Psychology, Universidad Loyola Andalucía, Seville. Spain.

(4) Multiple Sclerosis Unit. Hospital Universitario Virgen Macarena. Seville. Spain.

(5) Multiple Sclerosis Unit, Hospital Vithas. Seville. Spain.

***Supplementary Figure 1****.* Plotting of phase values in the lower and upper alpha and gamma bands for evoked and induced activities and in the cue interval following the presentation of a central cue and for each healthy control subject (26 subjects). Evoked activity is represented by red, yellow and green dots, and induced activity is displayed with green, blue and red crosses for lower alpha (8-10.5 Hz), upper alpha (10.5-13 Hz) and gamma (30-45 Hz) bands, respectively.


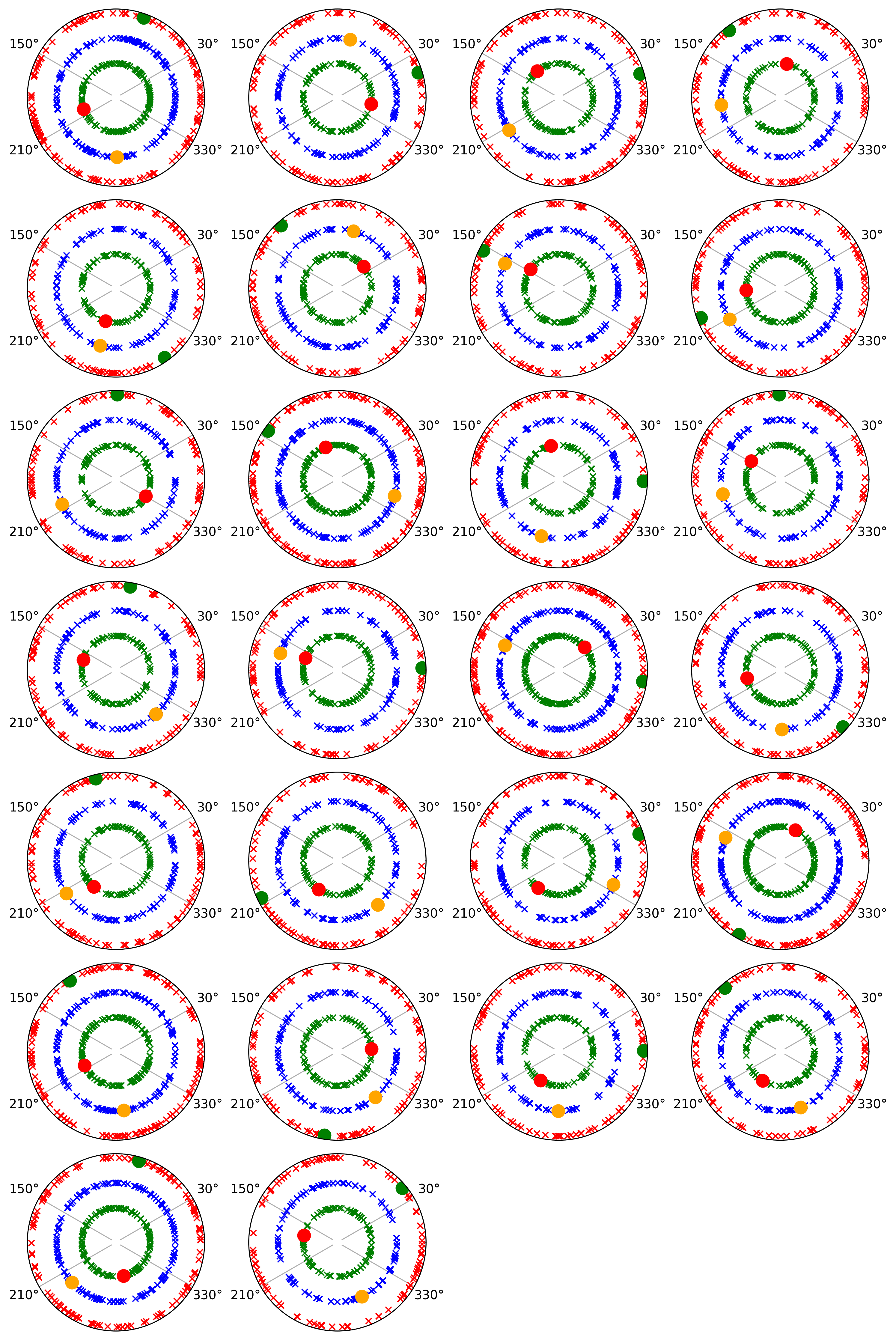


***Supplementary Figure 2.*** Plotting of phase values in the lower and upper alpha and gamma bands for evoked and induced activities and in the cue interval following the presentation of a spatial cue and for each healthy control subject (26 subjects). Evoked activity is represented by red, yellow and green dots, and induced activity is displayed with green, blue and red crosses for lower alpha (8-10.5 Hz), upper alpha (10.5-13 Hz) and gamma (30-45 Hz) bands, respectively. ***Supplementary Figure 3.*** Plotting of phase values in the lower and upper alpha and gamma bands for evoked and induced activities and in the target response interval following the presentation of a spatial cue and for each healthy control subject (26 subjects). Evoked activity is represented by red, yellow and green dots, and induced activity is displayed with green, blue and red crosses for lower alpha (8-10.5 Hz), upper alpha (10.5-13 Hz) and gamma (30-45 Hz) bands, respectively.


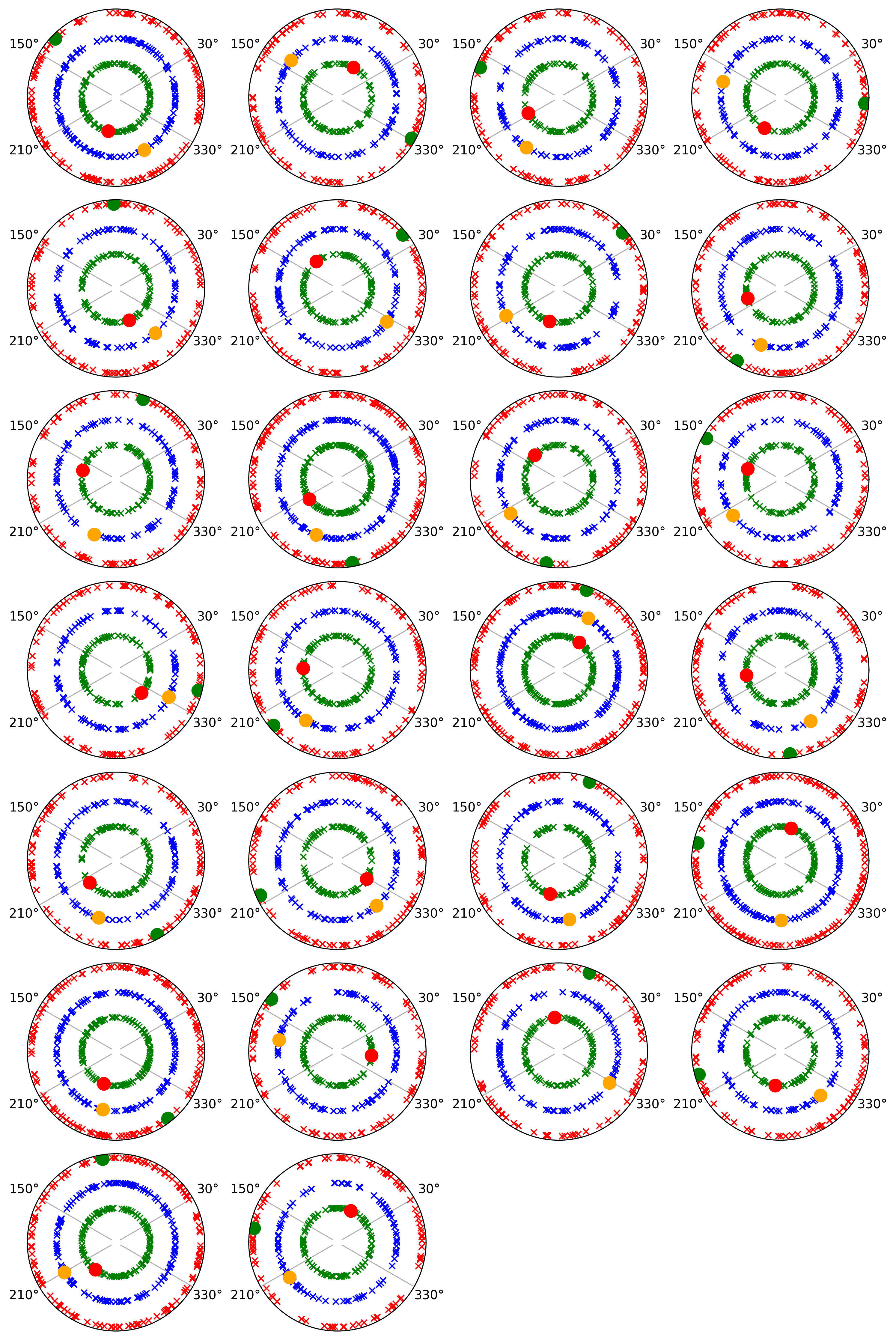

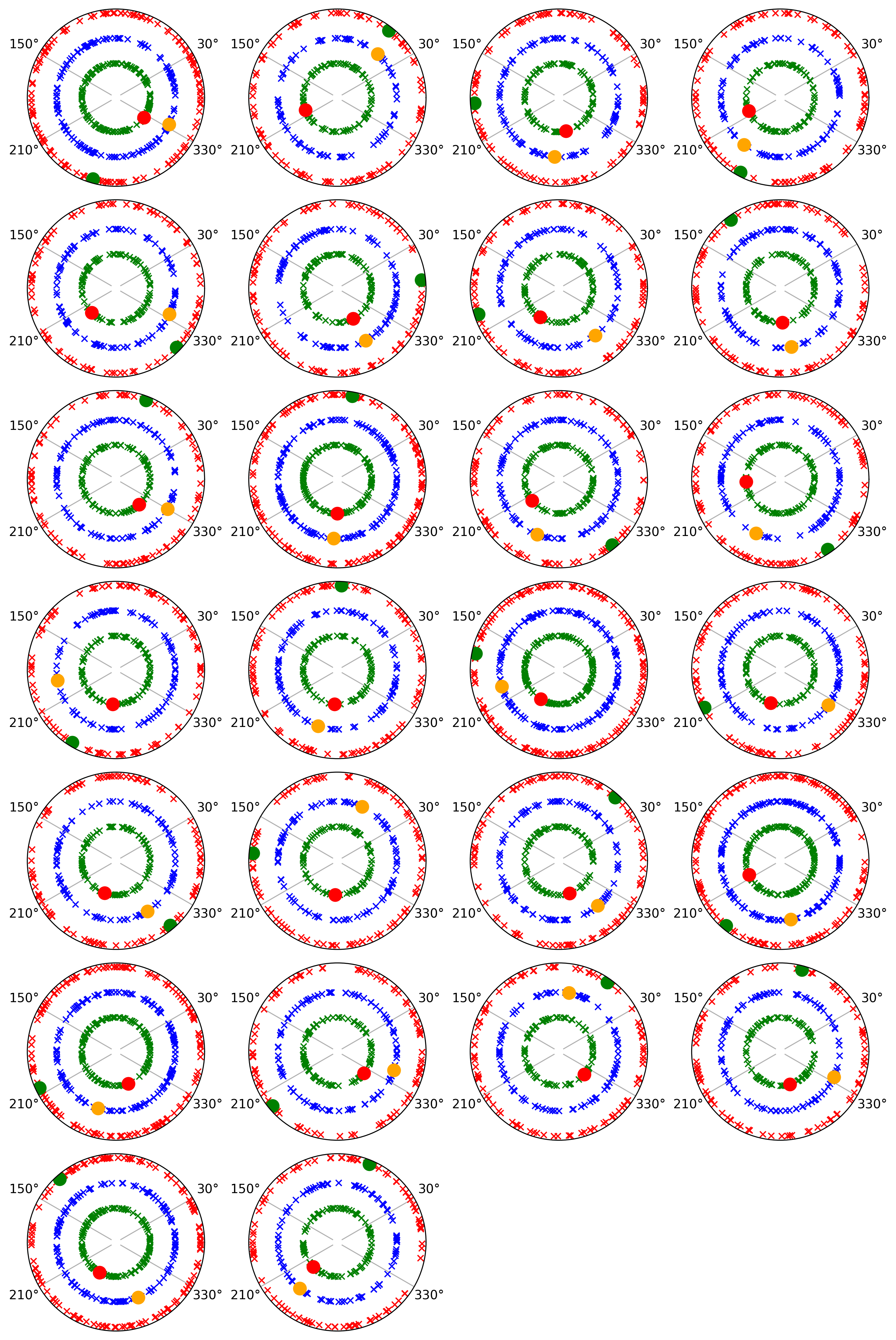


***Supplementary Figure 4.*** Plotting of phase values in the lower and upper alpha and gamma bands for evoked and induced activities and in the cue interval following the presentation of a central cue and for each MS subject (26 subjects). Evoked activity is represented by red, yellow and green dots, and induced activity is displayed with green, blue and red crosses for lower alpha (8-10.5 Hz), upper alpha (10.5-13 Hz) and gamma (30-45 Hz) bands, respectively.


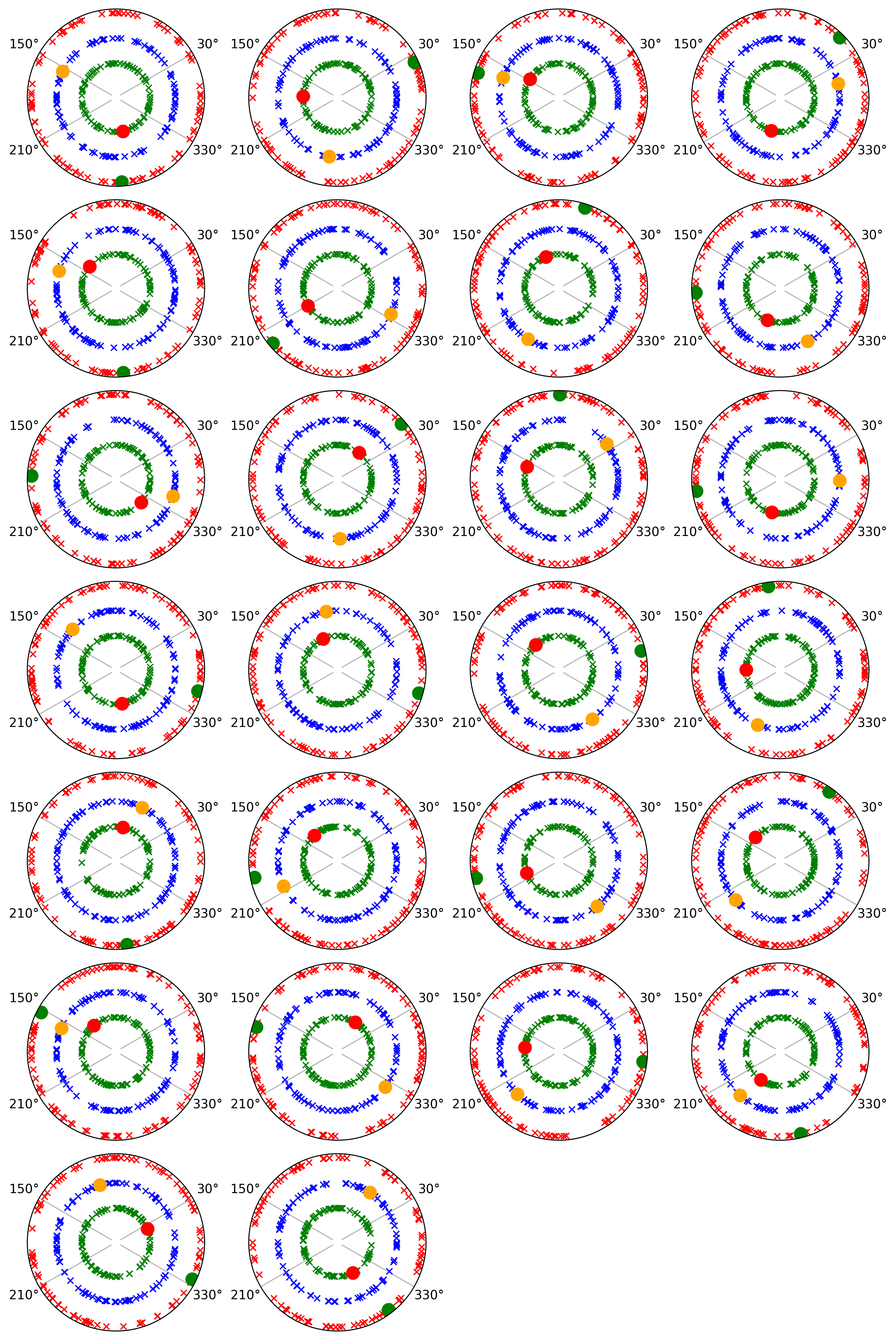


***Supplementary Figure 5.*** Plotting of phase values in the lower and upper alpha and gamma bands for evoked and induced activities and in the target response interval following the presentation of a central cue and for each MS subject (26 subjects). Evoked activity is represented by red, yellow and green dots, and induced activity is displayed with green, blue and red crosses for lower alpha (8-10.5 Hz), upper alpha (10.5-13 Hz) and gamma (30-45 Hz) bands, respectively.

***Supplementary Figure 6.*** Plotting of phase values in the lower and upper alpha and gamma bands for evoked and induced activities and in the cue interval following the presentation of a spatial cue and for each MS subject (26 subjects). Evoked activity is represented by red, yellow and green dots, and induced activity is displayed with green, blue and red crosses for lower alpha (8-10.5 Hz), upper alpha (10.5-13 Hz) and gamma (30-45 Hz) bands, respectively.


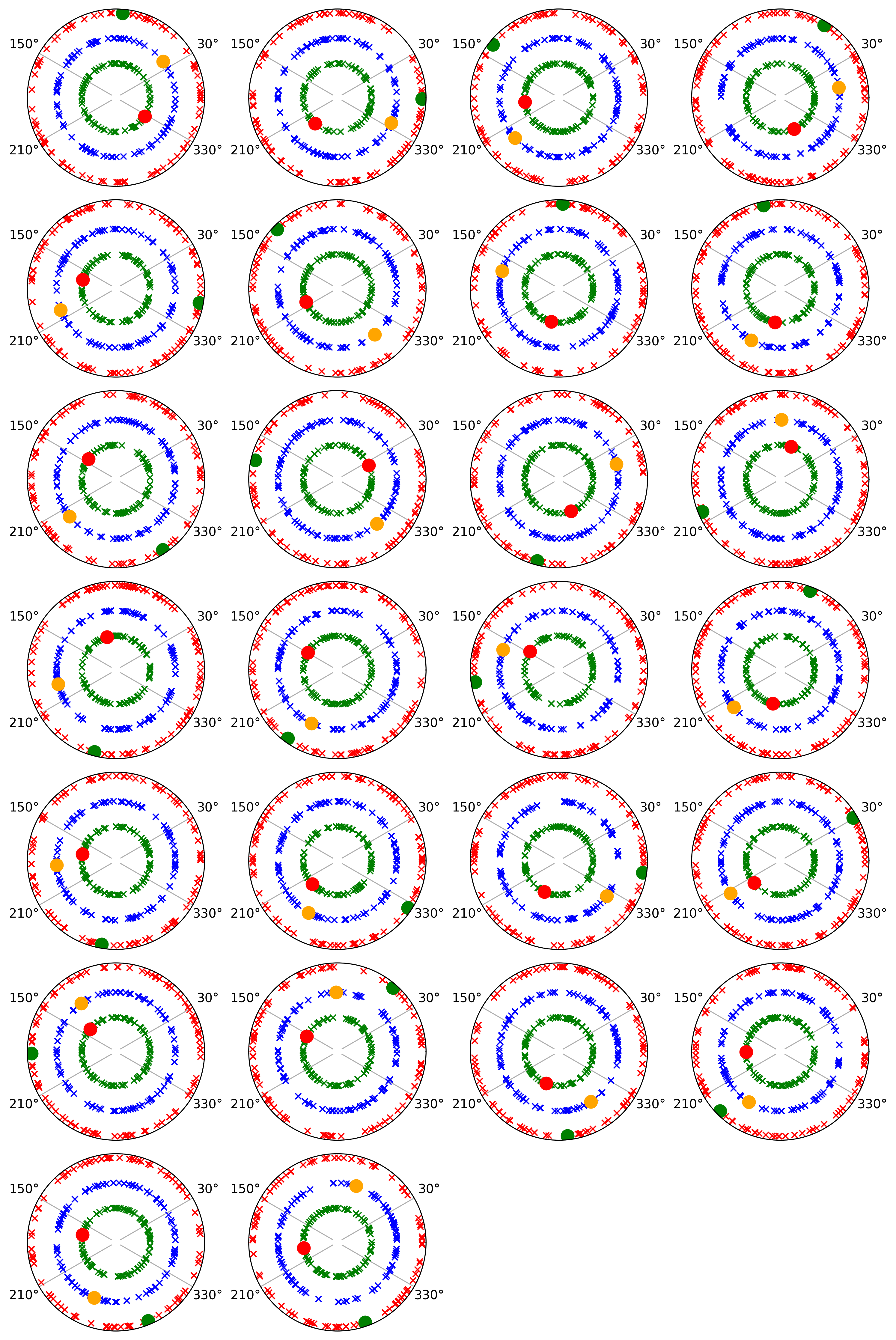

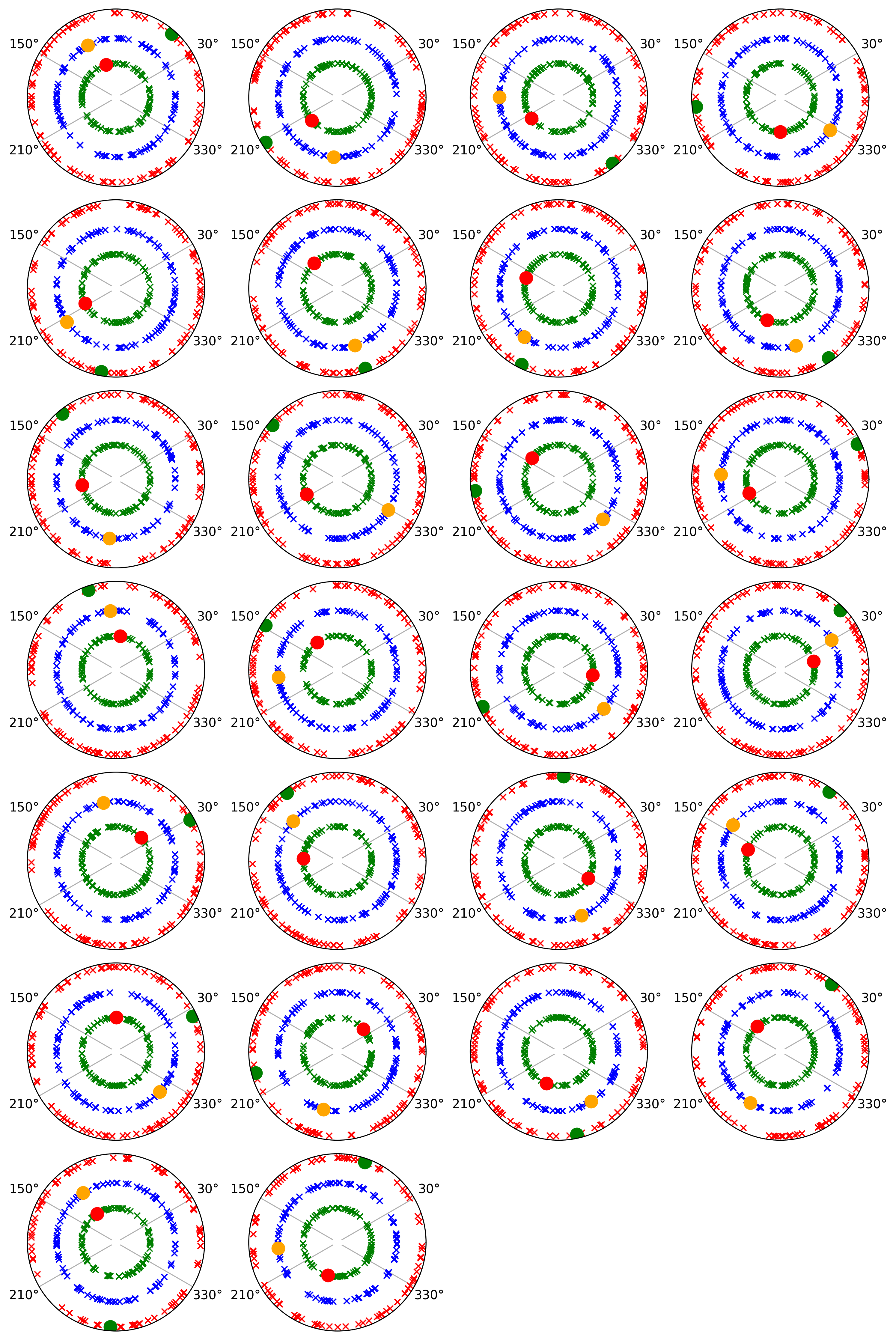


***Supplementary Figure 7.*** Plotting of phase values in the lower and upper alpha and gamma bands for evoked and induced activities and in the target response interval following the presentation of a spatial cue and for each MS subject (26 subjects). Evoked activity is represented by red, yellow and green dots, and induced activity is displayed with green, blue and red crosses for lower alpha (8-10.5 Hz), upper alpha (10.5-13 Hz) and gamma (30-45 Hz) bands, respectively.


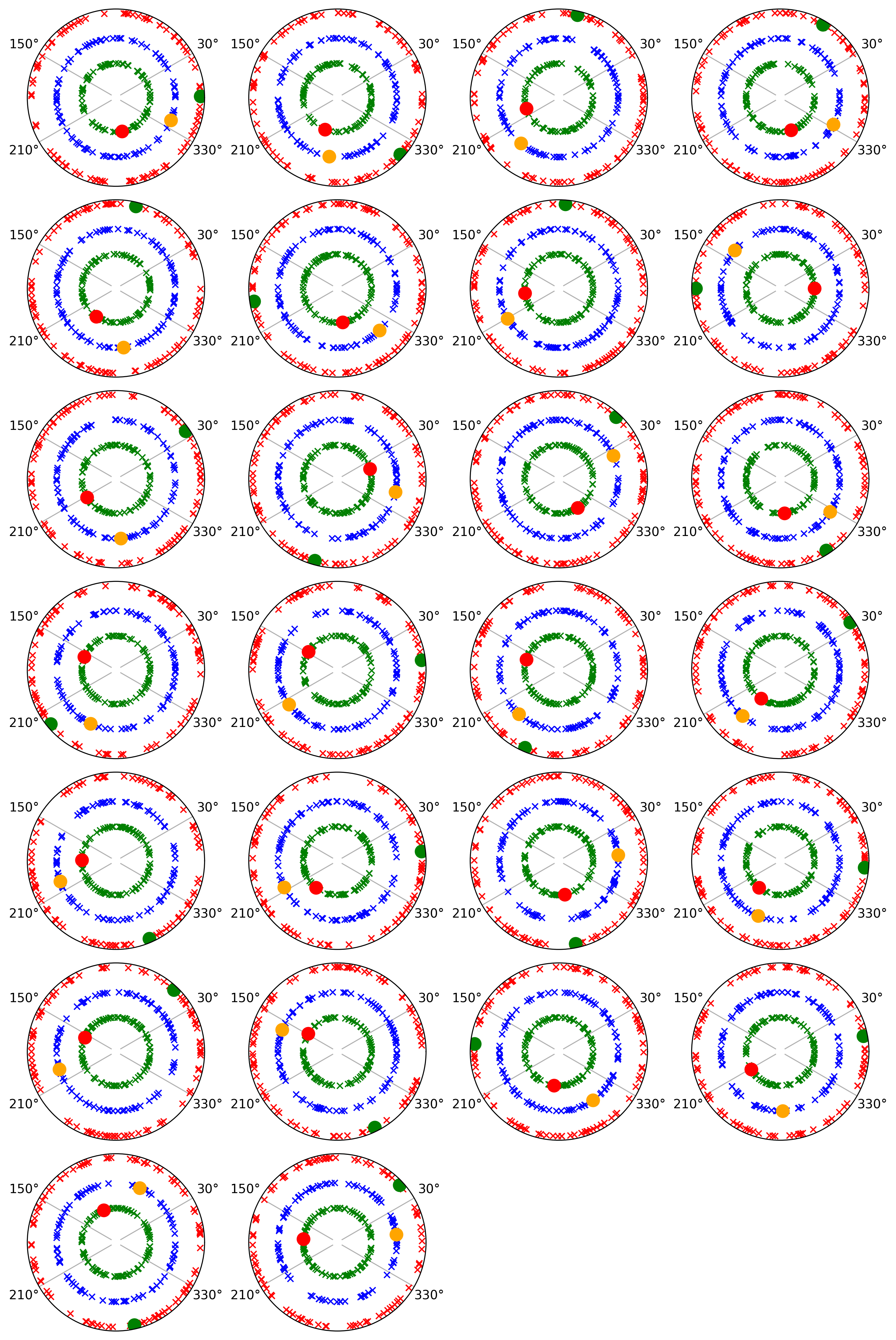


***Supplementary table 1.*** Statistical results for all the main factors and interactions between them.

1.1 Reaction time

| **ANOVA Reaction Time; HCs MS** | | |
| --- | --- | --- |
|  | **F** | **p** |
| **GROUP** | 26,551 | 0,000004 |
| **CUE** | 287,037 | 0,000000 |
| **CUE*GROUP** | 0,173 | 0,841129 |

HCs: Healthy Controls; MS: Multiple Sclerosis.

1.2 Reaction time (Fernández-Duque correction)

| **T-tests;HCsMS** | | | | |
| --- | --- | --- | --- | --- |
|  | **Mean - HCs** | **Mean - MS** | **t-value** | **p** |
| **NC** | 1,064880 | 1,052760 | 2,18627 | 0.033504 |
| **CC** | 1,009498 | 1,011457 | -0,35505 | 0,724042 |
| **SC** | 0,925623 | 0,935783 | -1,54650 | 0,128190 |

HCs: Healthy Controls; MS: Multiple Sclerosis; NC: No Cue; CC: Central Cue; SC: Spatial Cue.

1.3 Accuracy

| **Mann-Whitney U Test Precision; HCs MS** | | | | |
| --- | --- | --- | --- | --- |
|  | **Rank Sum - HCs** | **Rank Sum - MS** | **U** | **p-level** |
| **NC** | 730,0000 | 648,0000 | 297,0000 | 0,449384 |
| **CC** | 713,5000 | 664,5000 | 313,5000 | 0,652400 |
| **SC** | 727,0000 | 651,0000 | 300,0000 | 0,484210 |

HCs: Healthy Controls; MS: Multiple Sclerosis; NC: No Cue; CC: Central Cue; SC: Spatial Cue.

1.4 Latency

| **ANOVA Latency Expectancy Interval; HCs MS** | | |
| --- | --- | --- |
| **Gamma** | **F** | **p** |
| **Evoked Activity SC -GROUP** | 1,302 | 0,264657 |
| **Induced Activity SC - GROUP** | 1,956 | 0,174239 |
| **Low Alpha** | F | p |
| **Evoked Activity - GROUP** | 4,829 | 0,332781 |
| **Evoked Activity - CUE** | 3,239 | 0,289824 |
| **Evoked Activity - CUE*GROUP** | 1,726 | 0,355281 |
| **Induced Activity - GROUP** | 1,432 | 0,127281 |
| **Induced Activity - CUE** | 0,779 | 0,000000 |
| **Induced Activity - CUE*GROUP** | 1,193 | 0,097612 |
| **Upper Alpha** | F | p |
| **Evoked Activity - GROUP** | 2,3728 | 0,354762 |
| **Evoked Activity - CUE** | 5,2919 | 0,521922 |
| **Evoked Activity - CUE*GROUP** | 3,1280 | 0,472831 |
| **Induced Activity - GROUP** | 6,2863 | 0,185181 |
| **Induced Activity - CUE** | 6,3649 | 0,014421 |
| **Induced Activity - CUE*GROUP** | 4,9124 | 0,102739 |
| **ANOVA Latency Target Interval; HCs MS** | | |
| **Gamma** | **F** | **p** |
| **Evoked Activity - GROUP** | 7,2735 | 0,287361 |
| **Evoked Activity - CUE** | 5,9714 | 0,238102 |
| **Evoked Activity - CUE*GROUP** | 5,7413 | 0,662001 |
| **Induced Activity - GROUP** | 2,304 | 0,132110 |
| **Induced Activity - CUE** | 2,252 | 0,107805 |
| **Induced Activity - CUE*GROUP** | 0,271 | 0,762777 |
| **Low Alpha** | **F** | **p** |
| **Evoked Activity - GROUP** | 0,706 | 0,402842 |
| **Evoked Activity - CUE** | 1,636 | 0,197226 |
| **Evoked Activity - CUE*GROUP** | 0,722 | 0,487163 |
| **Induced Activity - GROUP** | 289,738 | 0,000000 |
| **Induced Activity - CUE** | 384,059 | 0,000000 |
| **Induced Activity - CUE*GROUP** | 81,212 | 0,000000 |
| **Upper Alpha** | **F** | **p** |
| **Evoked Activity - GROUP** | 0,105 | 0,746666 |
| **Evoked Activity - CUE** | 2,945 | 0,054862 |
| **Evoked Activity - CUE*GROUP** | 0,012 | 0,988042 |
| **Induced Activity - GROUP** | 163,726 | 0,000000 |
| **Induced Activity - CUE** | 360,851 | 0,000000 |
| **Induced Activity - CUE*GROUP** | 87,305 | 0,000000 |

HCs: Healthy Controls; MS: Multiple Sclerosis; SC: Spatial Cue.

1.5 Amplitude

| **ANOVA Amplitude Expectancy Interval** (210-225ms); HCs MS | | |
| --- | --- | --- |
| **Evoked Gamma** | **F** | **p** |
| **GROUP** | 1,057856 | 0,308655 |
| **CUE** | 3,856220 | 0,024362 |
| **CUE*GROUP** | 3,710018 | 0,027908 |
| **ANTPOST** | 0,096898 | 0,907734 |
| **ANTPOST*GROUP** | 1,989259 | 0,142174 |
| **LATMED** | 1,218112 | 0,296685 |
| **LATMED*GROUP** | 0,205321 | 0,974988 |
| **CUE*ANTPOST** | 2,496516 | 0,044060 |
| **CUE*ANTPOST*GROUP** | 1,408111 | 0,232595 |
| **CUE*LATMED** | 1,428833 | 0,147881 |
| **CUE*LATMED*GROUP** | 0,893507 | 0,553430 |
| **ANTPOST*LATMED** | 0,760508 | 0,691563 |
| **ANTPOST*LATMED*GROUP** | 0,558054 | 0,875865 |
| **CUE*ANTPOST*LATMED** | 0,698894 | 0,856670 |
| **CUE*ANTPOST*LATMED*GROUP** | 1,265466 | 0,176057 |
| **Induced Gamma** | **F** | **p** |
| **GROUP** | 4,51409 | 0,038583 |
| **CUE** | 53,89472 | 0,000000 |
| **CUE*GROUP** | 2,27933 | 0,107646 |
| **ANTPOST** | 0,70596 | 0,496079 |
| **ANTPOST*GROUP** | 1,25833 | 0,288587 |
| **LATMED** | 2,96064 | 0,008009 |
| **LATMED*GROUP** | 3,20621 | 0,004572 |
| **CUE*ANTPOST** | 0,55091 | 0,698568 |
| **CUE*ANTPOST*GROUP** | 1,76199 | 0,137959 |
| **CUE*LATMED** | 2,69410 | 0,001113 |
| **CUE*LATMED*GROUP** | 0,49854 | 0,915965 |
| **ANTPOST*LATMED** | 1,97301 | 0,024414 |
| **ANTPOST*LATMED*GROUP** | 1,88653 | 0,033214 |
| **CUE*ANTPOST*LATMED** | 0,94752 | 0,535570 |
| **CUE*ANTPOST*LATMED*GROUP** | 1,59919 | 0,033669 |

HCs: Healthy Controls; MS: Multiple Sclerosis; ANTPOST: Antero-posterior; LATMED: Lateral-medial.

| **ANOVA Amplitude Target Interval** (175-245ms); **HCs MS** | | |
| --- | --- | --- |
| **Evoked Gamma** | **F** | **p** |
| **GROUP** | 0,79968 | 0,375473 |
| **CUE** | 6,42172 | 0,002378 |
| **CUE*GROUP** | 0,39238 | 0,676486 |
| **CONGR** | 0,56768 | 0,454715 |
| **CONGR*GROUP** | 0,14554 | 0,704450 |
| **ANTPOST** | 7,42868 | 0,000982 |
| **ANTPOST*GROUP** | 1,77081 | 0,175490 |
| **LATMED** | 1,98471 | 0,067652 |
| **LATMED*GROUP** | 3,28618 | 0,003804 |
| **CUE*CONGR** | 0,32683 | 0,721977 |
| **CUE*CONGR*GROUP** | 0,37303 | 0,689601 |
| **CUE*ANTPOST** | 0,85853 | 0,489849 |
| **CUE*ANTPOST*GROUP** | 0,73455 | 0,569380 |
| **CONGR*ANTPOST** | 1,03459 | 0,359145 |
| **CONGR*ANTPOST*GROUP** | 0,54796 | 0,579857 |
| **CUE*LATMED** | 1,42065 | 0,151502 |
| **CUE*LATMED*GROUP** | 2,38286 | 0,005232 |
| **CONGR*LATMED** | 3,33401 | 0,003407 |
| **CONGR*LATMED*GROUP** | 0,33799 | 0,916496 |
| **ANTPOST*LATMED** | 2,14462 | 0,013005 |
| **ANTPOST*LATMED*GROUP** | 0,75624 | 0,695929 |
| **CUE*CONGR*ANTPOST** | 1,04225 | 0,386516 |
| **CUE*CONGR*ANTPOST*GROUP** | 0,32978 | 0,857744 |
| **CUE*CONGR*LATMED** | 0,70982 | 0,742611 |
| **CUE*CONGR*LATMED*GROUP** | 0,99008 | 0,456783 |
| **CUE*ANTPOST*LATMED** | 0,67049 | 0,883291 |
| **CUE*ANTPOST*LATMED*GROUP** | 0,63954 | 0,908797 |
| **CONGR*ANTPOST*LATMED** | 1,14993 | 0,316555 |
| **CONGR*ANTPOST*LATMED*GROUP** | 0,79903 | 0,651744 |
| **CUE*CONGR*ANTPOST*LATMED** | 1,02304 | 0,431806 |
| **CUE*CONGR*ANTPOST*LATMED*GROUP** | 0,90343 | 0,598053 |

HCs: Healthy Controls; MS: Multiple Sclerosis; ANTPOST: Antero-posterior; LATMED: Lateral-medial.

| **ANOVA Amplitude Target Interval** (175-245ms); **HCs MS** | | |
| --- | --- | --- |
| **Induced Gamma** | **F** | **p** |
| **GROUP** | 6,24882 | 0,015754 |
| **CUE** | 35,78958 | 0,000000 |
| **CUE*GROUP** | 3,03491 | 0,052530 |
| **CONGR** | 6,79198 | 0,012034 |
| **CONGR*GROUP** | 1,59302 | 0,212751 |
| **ANTPOST** | 4,29033 | 0,016307 |
| **ANTPOST*GROUP** | 0,01684 | 0,983309 |
| **LATMED** | 10,40524 | 0,000000 |
| **LATMED*GROUP** | 1,66054 | 0,130393 |
| **CUE*CONGR** | 3,67403 | 0,028860 |
| **CUE*CONGR*GROUP** | 1,26471 | 0,286799 |
| **CUE*ANTPOST** | 1,07266 | 0,371152 |
| **CUE*ANTPOST*GROUP** | 0,89910 | 0,465491 |
| **CONGR*ANTPOST** | 4,16739 | 0,018264 |
| **CONGR*ANTPOST*GROUP** | 0,59777 | 0,551991 |
| **CUE*LATMED** | 3,10230 | 0,000278 |
| **CUE*LATMED*GROUP** | 0,55273 | 0,879777 |
| **CONGR*LATMED** | 2,59844 | 0,018049 |
| **CONGR*LATMED*GROUP** | 0,05853 | 0,999195 |
| **ANTPOST*LATMED** | 1,64599 | 0,075147 |
| **ANTPOST*LATMED*GROUP** | 2,13113 | 0,013677 |
| **CUE*CONGR*ANTPOST** | 0,08695 | 0,986419 |
| **CUE*CONGR*ANTPOST*GROUP** | 0,27855 | 0,891623 |
| **CUE*CONGR*LATMED** | 0,67340 | 0,777780 |
| **CUE*CONGR*LATMED*GROUP** | 1,43055 | 0,147130 |
| **CUE*ANTPOST*LATMED** | 1,80758 | 0,009934 |
| **CUE*ANTPOST*LATMED*GROUP** | 1,63777 | 0,027116 |
| **CONGR*ANTPOST*LATMED** | 0,85896 | 0,589233 |
| **CONGR*ANTPOST*LATMED*GROUP** | 0,86550 | 0,582432 |
| **CUE*CONGR*ANTPOST*LATMED** | 0,91172 | 0,586299 |
| **CUE*CONGR*ANTPOST*LATMED*GROUP** | 1,29425 | 0,155325 |

HCs: Healthy Controls; MS: Multiple Sclerosis; ANTPOST: Antero-posterior; LATMED: Lateral-medial.

| **ANOVA Amplitude Expectancy Interval**; **HCs MS** | | |
| --- | --- | --- |
| **Evoked Low Alpha (0-350ms)** | **F** | **p** |
| **GROUP** | 7,1205 | 0,010247 |
| **CUE** | 34,2174 | 0,000000 |
| **CUE*GROUP** | 1,3267 | 0,269970 |
| **ANTPOST** | 29,3777 | 0,000000 |
| **ANTPOST*GROUP** | 2,6775 | 0,022289 |
| **LATMED** | 0,1198 | 0,993982 |
| **LATMED*GROUP** | 0,1714 | 0,984317 |
| **CUE*ANTPOST** | 10,0187 | 0,000000 |
| **CUE*ANTPOST*GROUP** | 0,5712 | 0,837836 |
| **CUE*LATMED** | 0,9556 | 0,490496 |
| **CUE*LATMED*GROUP** | 1,1776 | 0,295437 |
| **ANTPOST*LATMED** | 10,9617 | 0,000000 |
| **ANTPOST*LATMED*GROUP** | 0,7365 | 0,848950 |
| **CUE*ANTPOST*LATMED** | 2,7263 | 0,000000 |
| **CUE*ANTPOST*LATMED*GROUP** | 0,6689 | 0,976577 |
| **Induced Low Alpha (0-350ms)** | **F** | **p** |
| **GROUP** | 1,63999 | 0,206235 |
| **CUE** | 36,02478 | 0,000000 |
| **CUE*GROUP** | 0,06005 | 0,941750 |
| **ANTPOST** | 18,13771 | 0,000000 |
| **ANTPOST*GROUP** | 5,05864 | 0,000195 |
| **LATMED** | 26,26402 | 0,000000 |
| **LATMED*GROUP** | 0,09301 | 0,997011 |
| **CUE*ANTPOST** | 14,03191 | 0,000000 |
| **CUE*ANTPOST*GROUP** | 0,10880 | 0,999739 |
| **CUE*LATMED** | 0,91637 | 0,530001 |
| **CUE*LATMED*GROUP** | 0,67639 | 0,774954 |
| **ANTPOST*LATMED** | 3,55848 | 0,000000 |
| **ANTPOST*LATMED*GROUP** | 2,10541 | 0,000461 |
| **CUE*ANTPOST*LATMED** | 2,28266 | 0,000000 |
| **CUE*ANTPOST*LATMED*GROUP** | 0,87357 | 0,744620 |
| **Induced Low Alpha (350-700ms)** | **F** | **p** |
| **GROUP** | 0,50276 | 0,481584 |
| **CUE** | 19,25336 | 0,000000 |
| **CUE*GROUP** | 0,05944 | 0,942325 |
| **ANTPOST** | 17,47075 | 0,000000 |
| **ANTPOST*GROUP** | 1,61172 | 0,157408 |
| **LATMED** | 1,66277 | 0,129823 |
| **LATMED*GROUP** | 0,79748 | 0,572498 |
| **CUE*ANTPOST** | 14,57081 | 0,000000 |
| **CUE*ANTPOST*GROUP** | 0,44912 | 0,921602 |
| **CUE*LATMED** | 0,53199 | 0,894422 |
| **CUE*LATMED*GROUP** | 0,70730 | 0,745089 |
| **ANTPOST*LATMED** | 2,38785 | 0,000040 |
| **ANTPOST*LATMED*GROUP** | 0,79294 | 0,780059 |
| **CUE*ANTPOST*LATMED** | 1,04845 | 0,375480 |
| **CUE*ANTPOST*LATMED*GROUP** | 0,97480 | 0,531331 |

HCs: Healthy Controls; MS: Multiple Sclerosis; ANTPOST: Antero-posterior; LATMED: Lateral-medial.

| **ANOVA Amplitude Expectancy Interval**; **HCs MS** | | |
| --- | --- | --- |
| **Evoked Upper Alpha (0-350ms)** | **F** | **p** |
| **GROUP** | 4,47941 | 0,039306 |
| **CUE** | 28,24770 | 0,000000 |
| **CUE*GROUP** | 0,80449 | 0,450189 |
| **ANTPOST** | 23,35467 | 0,000000 |
| **ANTPOST*GROUP** | 0,91710 | 0,470426 |
| **LATMED** | 4,33795 | 0,000324 |
| **LATMED*GROUP** | 1,28398 | 0,264314 |
| **CUE*ANTPOST** | 7,64478 | 0,000000 |
| **CUE*ANTPOST*GROUP** | 0,88911 | 0,543224 |
| **CUE*LATMED** | 1,41892 | 0,152280 |
| **CUE*LATMED*GROUP** | 0,63611 | 0,811983 |
| **ANTPOST*LATMED** | 2,00766 | 0,001029 |
| **ANTPOST*LATMED*GROUP** | 1,14846 | 0,265865 |
| **CUE*ANTPOST*LATMED** | 0,97937 | 0,521312 |
| **CUE*ANTPOST*LATMED*GROUP** | 1,29987 | 0,061534 |
| **Induced Upper Alpha (0-350ms)** | **F** | **p** |
| **GROUP** | 0,05515 | 0,815288 |
| **CUE** | 33,08811 | 0,000000 |
| **CUE*GROUP** | 0,83554 | 0,436643 |
| **ANTPOST** | 7,62225 | 0,000001 |
| **ANTPOST*GROUP** | 0,78871 | 0,558643 |
| **LATMED** | 15,25929 | 0,000000 |
| **LATMED*GROUP** | 0,34031 | 0,915186 |
| **CUE*ANTPOST** | 18,36791 | 0,000000 |
| **CUE*ANTPOST*GROUP** | 0,08929 | 0,999895 |
| **CUE*LATMED** | 1,63204 | 0,078635 |
| **CUE*LATMED*GROUP** | 0,63790 | 0,810389 |
| **ANTPOST*LATMED** | 6,04353 | 0,000000 |
| **ANTPOST*LATMED*GROUP** | 2,88352 | 0,000000 |
| **CUE*ANTPOST*LATMED** | 2,35269 | 0,000000 |
| **CUE*ANTPOST*LATMED*GROUP** | 0,81342 | 0,847042 |
| **Induced Upper Alpha (350-700ms)** | **F** | **p** |
| **GROUP** | 0,00948 | 0,922840 |
| **CUE** | 19,74807 | 0,000000 |
| **CUE*GROUP** | 0,50472 | 0,605204 |
| **ANTPOST** | 20,91524 | 0,000000 |
| **ANTPOST*GROUP** | 1,27275 | 0,276167 |
| **LATMED** | 2,78349 | 0,011945 |
| **LATMED*GROUP** | 0,37878 | 0,892319 |
| **CUE*ANTPOST** | 17,37226 | 0,000000 |
| **CUE*ANTPOST*GROUP** | 0,32163 | 0,975435 |
| **CUE*LATMED** | 0,56808 | 0,868329 |
| **CUE*LATMED*GROUP** | 0,49926 | 0,915526 |
| **ANTPOST*LATMED** | 3,09823 | 0,000000 |
| **ANTPOST*LATMED*GROUP** | 0,58125 | 0,966105 |
| **CUE*ANTPOST*LATMED** | 0,97914 | 0,521816 |
| **CUE*ANTPOST*LATMED*GROUP** | 0,72111 | 0,947808 |

HCs: Healthy Controls; MS: Multiple Sclerosis; ANTPOST: Antero-posterior; LATMED: Lateral-medial.

| **ANOVA Amplitude Target Interval**; **HCs MS** | | |
| --- | --- | --- |
| **Evoked Low Alpha (0-350ms)** | **F** | **p** |
| **GROUP** | 8,4645 | 0,005392 |
| **CUE** | 26,6179 | 0,000000 |
| **CUE*GROUP** | 5,4573 | 0,005631 |
| **CONGR** | 32,5860 | 0,000001 |
| **CONGR*GROUP** | 5,2375 | 0,026367 |
| **ANTPOST** | 83,4161 | 0,000000 |
| **ANTPOST*GROUP** | 2,3203 | 0,103514 |
| **LATMED** | 10,8973 | 0,000000 |
| **LATMED*GROUP** | 0,3677 | 0,899112 |
| **CUE*CONGR** | 2,7030 | 0,071900 |
| **CUE*CONGR*GROUP** | 12,2045 | 0,000018 |
| **CUE*ANTPOST** | 40,4629 | 0,000000 |
| **CUE*ANTPOST*GROUP** | 0,1228 | 0,974172 |
| **CONGR*ANTPOST** | 15,6096 | 0,000001 |
| **CONGR*ANTPOST*GROUP** | 0,5074 | 0,603611 |
| **CUE*LATMED** | 8,6188 | 0,000000 |
| **CUE*LATMED*GROUP** | 1,3048 | 0,210966 |
| **CONGR*LATMED** | 1,7906 | 0,100650 |
| **CONGR*LATMED*GROUP** | 0,3879 | 0,886572 |
| **ANTPOST*LATMED** | 24,0749 | 0,000000 |
| **ANTPOST*LATMED*GROUP** | 0,7655 | 0,686403 |
| **CUE*CONGR*ANTPOST** | 1,7092 | 0,149350 |
| **CUE*CONGR*ANTPOST*GROUP** | 11,2802 | 0,000000 |
| **CUE*CONGR*LATMED** | 1,2020 | 0,277594 |
| **CUE*CONGR*LATMED*GROUP** | 1,3625 | 0,179393 |
| **CUE*ANTPOST*LATMED** | 8,8361 | 0,000000 |
| **CUE*ANTPOST*LATMED*GROUP** | 1,0148 | 0,442712 |
| **CONGR*ANTPOST*LATMED** | 4,5730 | 0,000000 |
| **CONGR*ANTPOST*LATMED*GROUP** | 0,8314 | 0,618005 |
| **CUE*CONGR*ANTPOST*LATMED** | 1,5736 | 0,038771 |
| **CUE*CONGR*ANTPOST*LATMED*GROUP** | 0,5119 | 0,975864 |
| **Induced Low Alpha (0-350ms)** | **F** | **p** |
| **GROUP** | 1,97357 | 0,166252 |
| **CUE** | 1,10607 | 0,334870 |
| **CUE*GROUP** | 3,19485 | 0,045188 |
| **CONGR** | 14,64141 | 0,000362 |
| **CONGR*GROUP** | 0,01007 | 0,920488 |
| **ANTPOST** | 60,03439 | 0,000000 |
| **ANTPOST*GROUP** | 2,67354 | 0,073939 |
| **LATMED** | 14,17310 | 0,000000 |
| **LATMED*GROUP** | 1,06857 | 0,381364 |
| **CUE*CONGR** | 5,45308 | 0,005652 |
| **CUE*CONGR*GROUP** | 0,75552 | 0,472429 |
| **CUE*ANTPOST** | 12,11219 | 0,000000 |
| **CUE*ANTPOST*GROUP** | 1,53793 | 0,192590 |
| **CONGR*ANTPOST** | 6,09140 | 0,003189 |
| **CONGR*ANTPOST*GROUP** | 0,37761 | 0,686474 |
| **CUE*LATMED** | 3,96625 | 0,000006 |
| **CUE*LATMED*GROUP** | 0,83941 | 0,609626 |
| **CONGR*LATMED** | 0,13468 | 0,991738 |
| **CONGR*LATMED*GROUP** | 1,13350 | 0,342670 |
| **ANTPOST*LATMED** | 4,67312 | 0,000000 |
| **ANTPOST*LATMED*GROUP** | 0,80740 | 0,643025 |
| **CUE*CONGR*ANTPOST** | 0,37175 | 0,828605 |
| **CUE*CONGR*ANTPOST*GROUP** | 0,77976 | 0,539523 |
| **CUE*CONGR*LATMED** | 0,96242 | 0,483771 |
| **CUE*CONGR*LATMED*GROUP** | 2,08227 | 0,016394 |
| **CUE*ANTPOST*LATMED** | 1,39313 | 0,098324 |
| **CUE*ANTPOST*LATMED*GROUP** | 1,75995 | 0,013272 |
| **CONGR*ANTPOST*LATMED** | 1,73402 | 0,056146 |
| **CONGR*ANTPOST*LATMED*GROUP** | 0,77858 | 0,672952 |
| **CUE*CONGR*ANTPOST*LATMED** | 0,60320 | 0,933976 |
| **CUE*CONGR*ANTPOST*LATMED*GROUP** | 0,62742 | 0,917767 |
| **Induced Low Alpha (350-700ms)** | **F** | **p** |
| **GROUP** | 0,82875 | 0,367003 |
| **CUE** | 7,68134 | 0,000788 |
| **CUE*GROUP** | 0,28374 | 0,753568 |
| **CONGR** | 30,98416 | 0,000001 |
| **CONGR*GROUP** | 0,00190 | 0,965395 |
| **ANTPOST** | 6,46369 | 0,002291 |
| **ANTPOST*GROUP** | 0,71430 | 0,492017 |
| **LATMED** | 2,20130 | 0,042862 |
| **LATMED*GROUP** | 1,30006 | 0,256858 |
| **CUE*CONGR** | 3,86166 | 0,024239 |
| **CUE*CONGR*GROUP** | 0,60037 | 0,550574 |
| **CUE*ANTPOST** | 3,53789 | 0,008169 |
| **CUE*ANTPOST*GROUP** | 0,93210 | 0,446312 |
| **CONGR*ANTPOST** | 0,64000 | 0,529440 |
| **CONGR*ANTPOST*GROUP** | 0,58746 | 0,557646 |
| **CUE*LATMED** | 2,92087 | 0,000595 |
| **CUE*LATMED*GROUP** | 0,67399 | 0,777224 |
| **CONGR*LATMED** | 1,68539 | 0,124163 |
| **CONGR*LATMED*GROUP** | 2,48696 | 0,023081 |
| **ANTPOST*LATMED** | 1,81051 | 0,043278 |
| **ANTPOST*LATMED*GROUP** | 0,87009 | 0,577661 |
| **CUE*CONGR*ANTPOST** | 0,06221 | 0,992813 |
| **CUE*CONGR*ANTPOST*GROUP** | 0,41955 | 0,794430 |
| **CUE*CONGR*LATMED** | 0,69224 | 0,759780 |
| **CUE*CONGR*LATMED*GROUP** | 0,91452 | 0,531882 |
| **CUE*ANTPOST*LATMED** | 0,91244 | 0,585268 |
| **CUE*ANTPOST*LATMED*GROUP** | 1,17556 | 0,254119 |
| **CONGR*ANTPOST*LATMED** | 1,73575 | 0,055820 |
| **CONGR*ANTPOST*LATMED*GROUP** | 0,87989 | 0,567498 |
| **CUE*CONGR*ANTPOST*LATMED** | 1,42216 | 0,085336 |
| **CUE*CONGR*ANTPOST*LATMED*GROUP** | 1,31477 | 0,141739 |

HCs: Healthy Controls; MS: Multiple Sclerosis; ANTPOST: Antero-posterior; LATMED: Lateral-medial.

| **ANOVA Amplitude Target Interval**; **HCs MS** | | |
| --- | --- | --- |
| **Evoked UpperAlpha (0-350ms)** | **F** | **p** |
| **GROUP** | 4,55027 | 0,037845 |
| **CUE** | 12,04810 | 0,000021 |
| **CUE*GROUP** | 0,35992 | 0,698632 |
| **CONGR** | 16,59620 | 0,000165 |
| **CONGR*GROUP** | 0,30495 | 0,583253 |
| **ANTPOST** | 65,33950 | 0,000000 |
| **ANTPOST*GROUP** | 1,32334 | 0,270867 |
| **LATMED** | 3,50047 | 0,002317 |
| **LATMED*GROUP** | 0,17749 | 0,982814 |
| **CUE*CONGR** | 1,97753 | 0,143787 |
| **CUE*CONGR*GROUP** | 2,36304 | 0,099371 |
| **CUE*ANTPOST** | 25,31502 | 0,000000 |
| **CUE*ANTPOST*GROUP** | 3,20852 | 0,013991 |
| **CONGR*ANTPOST** | 6,62644 | 0,001984 |
| **CONGR*ANTPOST*GROUP** | 0,82065 | 0,443086 |
| **CUE*LATMED** | 1,99667 | 0,022416 |
| **CUE*LATMED*GROUP** | 0,37783 | 0,971232 |
| **CONGR*LATMED** | 1,34475 | 0,237026 |
| **CONGR*LATMED*GROUP** | 0,93770 | 0,468132 |
| **ANTPOST*LATMED** | 12,58634 | 0,000000 |
| **ANTPOST*LATMED*GROUP** | 0,71384 | 0,738641 |
| **CUE*CONGR*ANTPOST** | 0,52244 | 0,719344 |
| **CUE*CONGR*ANTPOST*GROUP** | 0,65699 | 0,622604 |
| **CUE*CONGR*LATMED** | 1,07563 | 0,378066 |
| **CUE*CONGR*LATMED*GROUP** | 0,49298 | 0,919288 |
| **CUE*ANTPOST*LATMED** | 5,97056 | 0,000000 |
| **CUE*ANTPOST*LATMED*GROUP** | 0,85288 | 0,669068 |
| **CONGR*ANTPOST*LATMED** | 1,60270 | 0,086437 |
| **CONGR*ANTPOST*LATMED*GROUP** | 0,87648 | 0,571033 |
| **CUE*CONGR*ANTPOST*LATMED** | 0,50554 | 0,977775 |
| **CUE*CONGR*ANTPOST*LATMED*GROUP** | 0,44889 | 0,990217 |
| **Induced Upper Alpha (0-350ms)** | **F** | **p** |
| **GROUP** | 3,83570 | 0,055762 |
| **CUE** | 1,48022 | 0,232532 |
| **CUE*GROUP** | 2,35480 | 0,100155 |
| **CONGR** | 25,14632 | 0,000007 |
| **CONGR*GROUP** | 1,36408 | 0,248371 |
| **ANTPOST** | 56,46547 | 0,000000 |
| **ANTPOST*GROUP** | 3,45745 | 0,035325 |
| **LATMED** | 16,85691 | 0,000000 |
| **LATMED*GROUP** | 0,25393 | 0,957488 |
| **CUE*CONGR** | 2,90405 | 0,059437 |
| **CUE*CONGR*GROUP** | 4,19826 | 0,017751 |
| **CUE*ANTPOST** | 4,37736 | 0,002050 |
| **CUE*ANTPOST*GROUP** | 3,39979 | 0,010240 |
| **CONGR*ANTPOST** | 15,51153 | 0,000001 |
| **CONGR*ANTPOST*GROUP** | 0,55366 | 0,576593 |
| **CUE*LATMED** | 2,31160 | 0,006897 |
| **CUE*LATMED*GROUP** | 0,73846 | 0,714012 |
| **CONGR*LATMED** | 0,72695 | 0,628208 |
| **CONGR*LATMED*GROUP** | 1,08611 | 0,370623 |
| **ANTPOST*LATMED** | 5,31219 | 0,000000 |
| **ANTPOST*LATMED*GROUP** | 0,87903 | 0,568383 |
| **CUE*CONGR*ANTPOST** | 0,15418 | 0,960948 |
| **CUE*CONGR*ANTPOST*GROUP** | 0,93220 | 0,446257 |
| **CUE*CONGR*LATMED** | 1,19968 | 0,279266 |
| **CUE*CONGR*LATMED*GROUP** | 1,02717 | 0,421702 |
| **CUE*ANTPOST*LATMED** | 2,49708 | 0,000088 |
| **CUE*ANTPOST*LATMED*GROUP** | 1,05194 | 0,394264 |
| **CONGR*ANTPOST*LATMED** | 0,67269 | 0,778447 |
| **CONGR*ANTPOST*LATMED*GROUP** | 0,93083 | 0,515329 |
| **CUE*CONGR*ANTPOST*LATMED** | 0,32722 | 0,999177 |
| **CUE*CONGR*ANTPOST*LATMED*GROUP** | 0,39033 | 0,996563 |
| **Induced Upper Alpha (0-350ms)** | **F** | **p** |
| **GROUP** | 0,53664 | 0,467251 |
| **CUE** | 13,91944 | 0,000005 |
| **CUE*GROUP** | 0,64351 | 0,527605 |
| **CONGR** | 11,29087 | 0,001498 |
| **CONGR*GROUP** | 0,11288 | 0,738295 |
| **ANTPOST** | 3,92798 | 0,022793 |
| **ANTPOST*GROUP** | 0,85797 | 0,427121 |
| **LATMED** | 4,71511 | 0,000132 |
| **LATMED*GROUP** | 0,86856 | 0,518312 |
| **CUE*CONGR** | 1,31147 | 0,274016 |
| **CUE*CONGR*GROUP** | 2,23204 | 0,112629 |
| **CUE*ANTPOST** | 6,31916 | 0,000083 |
| **CUE*ANTPOST*GROUP** | 0,61886 | 0,649569 |
| **CONGR*ANTPOST** | 3,67918 | 0,028721 |
| **CONGR*ANTPOST*GROUP** | 0,24123 | 0,786116 |
| **CUE*LATMED** | 4,44169 | 0,000001 |
| **CUE*LATMED*GROUP** | 0,61537 | 0,830063 |
| **CONGR*LATMED** | 2,22733 | 0,040541 |
| **CONGR*LATMED*GROUP** | 1,23946 | 0,285870 |
| **ANTPOST*LATMED** | 2,23513 | 0,009244 |
| **ANTPOST*LATMED*GROUP** | 0,90642 | 0,540166 |
| **CUE*CONGR*ANTPOST** | 0,23133 | 0,920538 |
| **CUE*CONGR*ANTPOST*GROUP** | 0,37621 | 0,825454 |
| **CUE*CONGR*LATMED** | 0,31780 | 0,986262 |
| **CUE*CONGR*LATMED*GROUP** | 0,97879 | 0,467715 |
| **CUE*ANTPOST*LATMED** | 1,07278 | 0,368195 |
| **CUE*ANTPOST*LATMED*GROUP** | 1,31765 | 0,139906 |
| **CONGR*ANTPOST*LATMED** | 1,21272 | 0,270025 |
| **CONGR*ANTPOST*LATMED*GROUP** | 0,97380 | 0,472586 |
| **CUE*CONGR*ANTPOST*LATMED** | 0,72781 | 0,826565 |
| **CUE*CONGR*ANTPOST*LATMED*GROUP** | 1,08080 | 0,358399 |

HCs: Healthy Controls; MS: Multiple Sclerosis; ANTPOST: Antero-posterior; LATMED: Lateral-medial.

***Supplementary table 2.*** Post hoc amplitude values for the cue x anterior-posterior location x medial-lateral position interaction.

| **Gamma**  **Expectancy Interval** | | | |  |
| --- | --- | --- | --- | --- |
|  | NC | CC | SC |  |
| Electrode | Induced | Induced | Induced | |
| CP5 | -0.0063 | -0.0113 | 0.25357 | |
| CP3 | -0.0084 | -0.0258 | 0.28823 | |
| CP1 | 0.00437 | -0.0128 | 0.34615 | |
| CPz | 0.0038 | -0.0202 | 0.35917 | |
| CP2 | 0.00183 | -0.0016 | 0.34863 | |
| CP4 | 0.00723 | 0.01504 | 0.32981 | |
| CP6 | -0.0010 | 0.00003 | 0.27958 | |
| P5 | -0.0002 | -0.0521 | 0.29932 | |
| P3 | 0.01728 | -0.0154 | 0.29774 | |
| P1 | 0.00387 | -0.0103 | 0.33840 | |
| Pz | 0.00458 | -0.0114 | 0.34338 | |
| P2 | 0.00633 | -0.0006 | 0.36236 | |
| P4 | 0.01282 | -0.0031 | 0.35489 | |
| P6 | -0.0033 | -0.0126 | 0.31683 | |
| PO5 | 0.01585 | -0.0075 | 0.29987 | |
| PO3 | 0.00463 | -0.0081 | 0.32278 | |
| PO1 | 0.00317 | -0.0191 | 0.34413 | |
| Pzp | 0.01234 | -0.0189 | 0.33406 | |
| PO2 | 0.00324 | -0.0189 | 0.32406 | |
| PO4 | -0.0045 | -0.130 | 0.32588 | |
| PO6 | 0.00863 | 0.00314 | 0.30794 | |

| **Gamma**  **Target Interval** | | | | | | |
| --- | --- | --- | --- | --- | --- | --- |
|  | NC | | CC | | SC | |
| Electrode | Evoked | Induced | Evoked | Induced | Evoked | Induced |
| CP5 | 0.03667 | 0.32638 | 0.06452 | 0.27602 | 0.00808 | 0.09088 |
| CP3 | 0.04976 | 0.35857 | 0.07841 | 0.34011 | 0.01443 | 0.10979 |
| CP1 | 0.05829 | 0.37645 | 0.08069 | 0.38736 | 0.01978 | 0.12428 |
| CPz | 0.06508 | 0.39475 | 0.08518 | 0.40448 | 0.02511 | 0.12860 |
| CP2 | 0.06943 | 0.39011 | 0.08886 | 0.38859 | 0.02794 | 0.11650 |
| CP4 | 0.06425 | 0.37061 | 0.07375 | 0.36901 | 0.02627 | 0.12029 |
| CP6 | 0.05208 | 0.30946 | 0.05329 | 0.31218 | 0.03053 | 0.09462 |
| P5 | 0.06483 | 0.36669 | 0.07882 | 0.33145 | 0.02824 | 0.12025 |
| P3 | 0.06655 | 0.35030 | 0.08945 | 0.37734 | 0.02664 | 0.13731 |
| P1 | 0.06757 | 0.39667 | 0.08840 | 0.40472 | 0.02704 | 0.13335 |
| Pz | 0.06752 | 0.37930 | 0.08519 | 0.38956 | 0.02361 | 0.12527 |
| P2 | 0.07632 | 0.41087 | 0.08902 | 0.41026 | 0.02918 | 0.13575 |
| P4 | 0.07244 | 0.39572 | 0.08012 | 0.40168 | 0.03332 | 0.12518 |
| P6 | 0.06338 | 0.34715 | 0.07108 | 0.36150 | 0.01610 | 0.12847 |
| PO5 | 0.07236 | 0.35552 | 0.08411 | 0.35576 | 0.02603 | 0.11132 |
| PO3 | 0.07115 | 0.39416 | 0.09799 | 0.38565 | 0.02759 | 0.10994 |
| PO1 | 0.07384 | 0.40154 | 0.08875 | 0.41171 | 0.02877 | 0.13448 |
| Pzp | 0.06840 | 0.39454 | 0.09069 | 0.40446 | 0.02303 | 0.13889 |
| PO2 | 0.06887 | 0.38460 | 0.08636 | 0.39271 | 0.02096 | 0.12531 |
| PO4 | 0.07271 | 0.36272 | 0.08056 | 0.36702 | 0.02339 | 0.11885 |
| PO6 | 0.07053 | 0.33909 | 0.06810 | 0.32838 | 0.02664 | 0.10002 |

***Supplementary table 3.*** Phase values for evoked and induced activity for all bands and experimental conditions. See supplementary Excel file termed “Phase values”.
